# Trapping *para*-Quinone Methide Intermediates with Ferrocene: Synthesis and Preliminary Biological Evaluation of New Phenol-Ferrocene Conjugates

**DOI:** 10.3390/molecules23061335

**Published:** 2018-06-01

**Authors:** Silvia González-Pelayo, Enol López, Javier Borge, Noemí de-los-Santos-Álvarez, Luis A. López

**Affiliations:** 1 Departamento de Química Orgánica e Inorgánica and Instituto Universitario de Química Organometálica “Enrique Moles”, Universidad de Oviedo, Julián Clavería 8, 33006 Oviedo, Spain; gonzalezpelayos@gmail.com (S.G.-P.); enol.lopezh@gmail.com (E.L.); 2 Laboratorio de Compuestos Organometálicos y Catálisis (Unidad Asociada al CSIC), Centro de Innovación en Química Avanzada (ORFEO-CINQA) and Departamento de Química Física y Analítica, Universidad de Oviedo, Julián Clavería 8, 33006 Oviedo, Spain; jborge@uniovi.es; 3 Departamento de Química Física y Analítica, Universidad de Oviedo, Julián Clavería 8, 33006 Oviedo, Spain; santosnoemi@uniovi.es

**Keywords:** ferrocene, phenol, *para*-quinone methides, cytotoxic activity

## Abstract

The reaction of *para*-hydroxybenzyl alcohols with ferrocene in the presence of a catalytic amount of InCl_3_ provided ferrocenyl phenol derivatives, an interesting class of organometallic compounds with potential applications in medicinal chemistry. This transformation exhibited a reasonable substrate scope delivering the desired products in synthetically useful yields. Evidence of involvement of a *para*-quinone methide intermediate in this coupling process was also provided. Preliminary biological evaluation demonstrated that some of the ferrocene derivatives available by this methodology exhibit significant cytotoxicity against several cancer cell lines with IC_50_ values within the range of 1.07–4.89 μM.

## 1. Introduction

**Figure 1:**
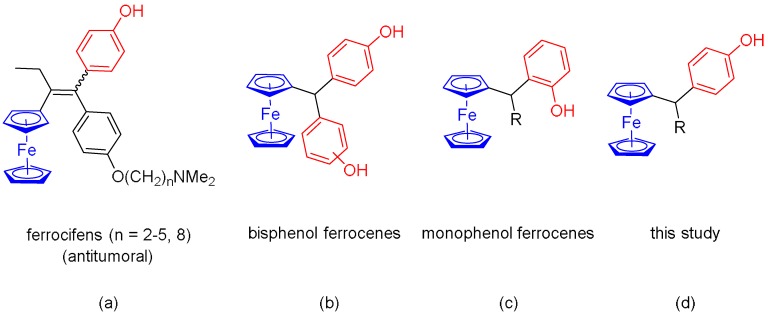
Phenol-ferrocene conjugates: (**a**) Ferrocifen family; (**b**) ferrocene bisphenol derivatives; (**c**) *ortho*-substituted ferrocenyl phenols previously developed in our group; (**d**) *para*-substituted ferrocenyl phenols reported in this study.

Since the discovery of ferrocene in the 1950s [1,2], the interest in this organometallic compound has not declined. In fact, its chemistry remains one of the most active areas of research. Very likely, this enduring interest resides in the fact that many functionalized ferrocene derivatives display a wide number of applications in a diverse range of fields [3,4,5,6,7,8,9]. For example, recent investigations have demonstrated the potential of some ferrocene derivatives in medicinal chemistry [10,11]. Particularly, some ferrocene-containing phenols have proved to be of great interest in cancer therapeutics because of their antitumoral activity [12,13,14,15,16]. Among them, a family of ferrocene analogues of hydroxytamoxifen, the so-called ferrocifens (Figure 1a), have been the subject of in-depth investigations showing exceptional cytotoxic activities against some types of breast cancer [17,18,19]. The mode of action of these organometallic drug candidates has been elucidated by electrochemical and chemical oxidation methods. According to these studies, ferrocenyl quinone methides have been suggested to play a key role in the antiproliferative activity [20,21,22,23,24]. The antitumoral activity of some unconjugated bisphenol derivatives of ferrocene (Figure 1b) has also been evaluated [25,26].

In connection with our studies on C–H bond functionalization of ferrocene based on the trapping of highly electrophilic species [27,28,29], we have recently described the trapping of *ortho*-quinone methide intermediates with ferrocene [30]. Interestingly, some of the ferrocene-containing monophenol derivatives available by this methodology (Figure 1c) display remarkable cytotoxic activity against various cancer cell lines.

In order to elucidate the structural requirements for cytotoxicity and, eventually, to identify more bioactive derivatives, we decided to develop a synthetic methodology for the synthesis of the isomeric *para*-substituted ferrocenylphenol analogues (Figure 1d). Herein, we report the results of this study; specifically, we describe the generation of *para*-quinone methide intermediates and their trapping with ferrocene. Preliminary biological evaluation of some of the functionalized ferrocene derivatives prepared in this study is also disclosed.

## 2. Results and Discussion

**Figure 2:**
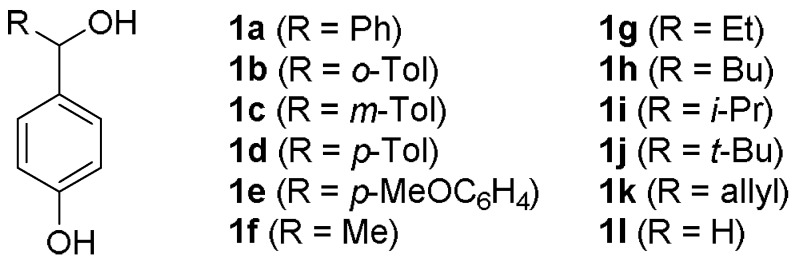
Starting *p*-hydroxybenzyl alcohols **1** used in this work.

The present study was carried out using easily available *p*-hydroxybenzyl alcohols **1a**–**l** outlined in Figure 2.

**Scheme 1:**
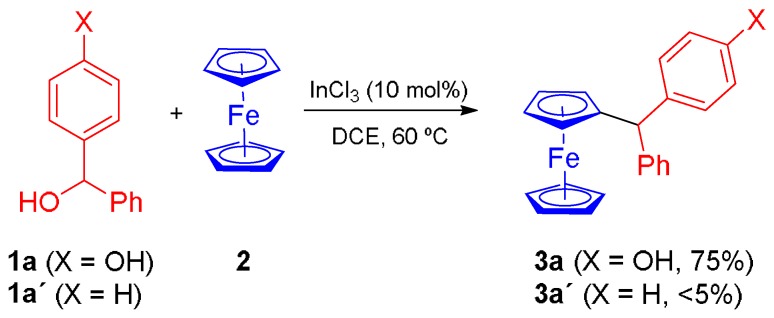
Trapping of *p*-quinone methides with ferrocene: proof of concept. DCE: dichloroethane.

For our initial study, *p*-hydroxybenzyl alcohol **1a** was chosen as the model substrate (Scheme 1). On the basis of our previous investigations in the *ortho* series, InCl_3_ in dichloroethane (DCE) was selected as the catalytic system. Pleasingly, we found that heating a mixture of **1a** (1 equiv.), ferrocene (**2**, 3 equiv.), and InCl_3_ (10 mol%) in DCE at 60 °C led to complete disappearance of the starting *p*-hydroxybenzyl alcohol after 2 h (checked by thin layer chromatography, TLC). Chromatographic purification (SiO_2_, 5:1 hexane ethyl acetate) provided the desired functionalized ferrocene derivative **3a** in a remarkable 75% yield. Interestingly, under otherwise similar conditions, benzydryl alcohol **1a’** was found to be a fruitless reaction partner, thus demonstrating the key role of the phenolic OH group in the reaction course [31,32].

**Figure 3:**
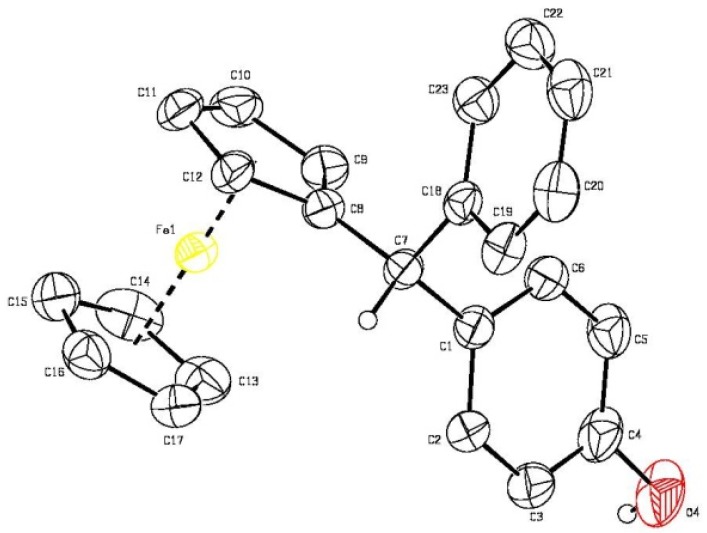
X-ray structure of ferrocene derivative **3a**. Thermal ellipsoids are drawn at the 30% probability level. Hydrogen atoms are excluded, except those bonded to C7 (H7) and O4 (H4O).

Ferrocenyl phenol **3a** was characterized by Nuclear Magnetic Resonance (NMR) spectroscopy. Moreover, crystals of compound **3a** were obtained from diffusion of pentane into a dichloromethane solution at −20 °C and its molecular structure in the solid state has been determined by single-crystal X-ray diffraction (Figure 3 and Appendix A) [33,34]. The electrochemical behavior of compound **3a** was investigated by cyclic voltammetry (Appendix B).

With suitable reaction conditions in hand (10 mol% of InCl_3_, DCE as solvent, 60 °C), a variety of *p*-hydroxybenzyl alcohols were then evaluated for their suitability for this C–H bond functionalization process (Table 1). First, some *p*-hydroxybenzyl alcohols substituted with various aryl groups were investigated (entries 2–5). As shown, all three isomeric 4-[hydroxy(tolyl)methyl]phenol derivatives **1b**–**d** (R = tolyl) served as suitable reaction partners for this process furnishing the desired functionalized ferrocene derivatives **3b**–**d** in acceptable isolated yields (51–82%, entries 2–4). Similarly, *para*-methoxy substituted substrate **1e** (R = *p*-MeOC_6_H_4_) delivered the corresponding product **3e** in moderate isolated yield (48%, entry 5).

Next, substrates **1f**–**j** with alkyl groups in the benzylic position were tested (entries 6–10). As shown, primary, secondary and tertiary alkyl groups were well tolerated delivering functionalized ferrocene derivatives **3f**–**j** in moderate yields (42–62%).

This transformation was compatible with unsaturated functional groups in the benzylic position as demonstrated by the synthesis of ferrocene derivative **3k** in moderate yield when substrate **1k** (R = allyl) was subjected to the standard reaction conditions. Finally, we found that the parent *p*-hydroxybenzyl alcohol **1l** (R = H) is also a viable substrate affording the desired product **3l** in 64% yield (entry 12).

**Scheme 2:**
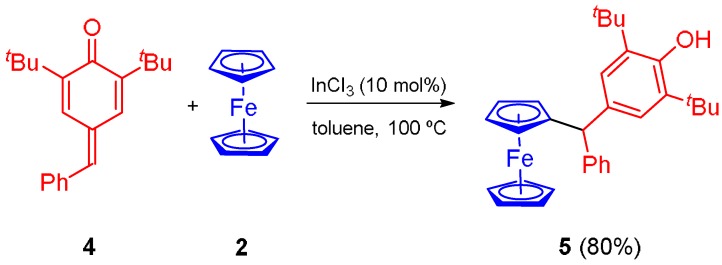
InCl_3_-catalyzed reaction of ferrocene and stable *p*-quinone methide **4**.

Next, to provide further evidence for the involvement of *p*-quinone methide intermediates in the present coupling, we performed an experiment with a stable *p*-quinone methide. Thus, 4-benzylidene-2,6-di-*tert*-butylcyclohexa-2,5-dienone (**4**) and ferrocene (**2**) were subjected to the standard reaction conditions (10 mol% of InCl_3_, DCE, 60 °C). However, a very low conversion was observed after 24 h very likely due to steric hindrance by the *tert*-butyl groups. Gratifyingly, performing the reaction in toluene at 100 °C enabled the preparation of ferrocene derivative **5** in 80% yield (Scheme 2). Notably, in the absence of InCl_3_, no reaction occurred at all.

**Scheme 3:**
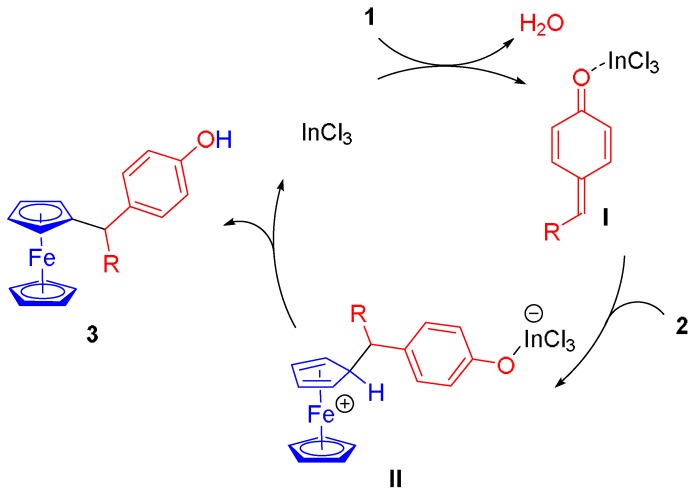
Mechanism for the InCl_3_-catalyzed reaction of phenol derivatives **1** and ferrocene (**2**).

Based on these control experiments and on previous related literature precedents, a mechanistic proposal for the reaction of hydroxybenzyl alcohols **1** and ferrocene (**2**) is outlined in Scheme 3. In the present process, the Lewis acid is proposed to play a dual role. Firstly, it would promote the generation of the key quinone methide intermediate through dehydration of hydroxybenzyl alcohol **1**. Subsequent activation of the quinone methide by Lewis acid complexation would provide intermediate **I**. This intermediate, with a high electrophilic character at the exocyclic C=C bond, may be involved in a Friedel-Crafts type electrophilic aromatic substitution [35]. Indeed, 1,6-addition of ferrocene would provide the *σ*-complex intermediate **II**, which would evolve to the final product with regeneration of the catalyst [36].

Some of the functionalized ferrocene derivatives prepared were evaluated for their cytotoxicity against several cancer cell lines (Table 2). In this preliminary study, ferrocene derivatives **3a** and **3g** were identified as the most active ones [37]. For example, **3a** displayed significant toxicity against A2780 ovarian cancer cell line (IC_50_ of 1.07 μM). Compared with the value previously reported for the *ortho*-isomer (IC_50_ of 2.68 μM), ferrocene derivative **3a** has superior characteristics. Ferrocene **3g** also displayed toxicity against this cell line (IC_50_ of 2.23 μM), although somewhat lower than that found for the *ortho*-analogue (IC_50_ of 1.86 μM). We have also studied the cytotoxicity profile of ferrocene derivatives **3a** and **3g** against A549 lung cancer cells. Both derivatives exhibited moderate cytotoxicity with IC_50_ values of 3.55 and 4.89 μM, respectively. These values are comparable to that previously measured for the *ortho*-isomers (IC_50_ of 2.77 and 5.96 μM, respectively).

## 3. Materials and Methods

### 3.1. General

NMR spectra were recorded at room temperature in CDCl_3_ on a Bruker DPX-300 or Bruker AVANCE-300 MHz instruments (Bruker, Billerica, MA, USA). Chemical shifts are given in ppm relative to TMS (^1^H, 0.0 ppm) or CDCl_3_ (^13^C, 77.0 ppm). High-resolution mass spectra were determined on a VG Autospec M mass spectrometer (Waters Corporation, Milford, MA, USA). Cyclic voltammetric studies were performed using a µ-AutoLab type II equipped with GPES 4.9 software (EcoChemie, Utrecht, The Netherlands). All measurements were carried out using a conventional three electrode system in phosphate saline buffer (pH 7.4). A modified carbon paste acted as the working electrode and a Pt wire as a counter electrode. All potentials were referred to a Ag|AgCl|KCl_(sat)_ reference electrode.

Experiments were carried out under nitrogen using standard Schlenck techniques. 1,2-Dichloroethane was distilled from CaH_2_. Toluene was distilled from sodium-benzophenone ketyl prior to use. TLC was performed on aluminum-backed plates coated with silica gel 60 with F_254_ indicator. Flash column chromatography was carried out on silica gel (230–240 mesh). The solvents used in column chromatography, hexane and ethyl acetate, were obtained from commercial suppliers and used without further purification.

*p*-Hydroxybenzyl alcohols **1a**–**k** were prepared by reaction of 4-hydroxybenzaldehyde with the corresponding Grignard reagents following a literature procedure [38]. *p*-Hydroxybenzyl alcohol **1l** was obtained by the reaction of 4-hydroxybenzaldehyde with NaBH_4_ [39]. 4-Benzylidene-2,6-di-*tert*-butylcyclohexa-2,5-dienone (**4**) was prepared from 2,6-di-*tert*-butylphenol and benzaldehyde according to a literature procedure [40]. Ferrocene (**2**) was commercially available and used without further purification.

### 3.2. General Procedure for the Synthesis of Ferrocene Derivatives ***3a**–**l***

InCl_3_ (4.4 mg, 0.02 mmol, 10 mol%) was added to a solution of *p*-hydroxybenzyl alcohols **1** (0.2 mmol) and ferrocene **2** (111.6 mg, 0.6 mmol) in 1,2-dichloroethane (2 mL). The mixture was stirred at 60 °C for 2–14 h (disappearance of **1** checked by TLC). The solvent was removed under reduced pressure and the resulting residue was purified by flash chromatography (silica gel, mixtures of hexanes/ethyl acetate). Two fractions were collected. The first fraction was unreacted ferrocene and the second one was the corresponding functionalized ferrocene derivative **3**. Crystals of compound **3a** suitable for X-ray analysis were obtained from diffusion of pentane into dichloromethane at −20 °C. Copies of ^1^H- and ^13^C-NMR spectra are provided in the Supplementary Materials.

*4-[(Ferrocenyl)(phenyl)methyl]phenol* (**3a**): yellow solid; melting point (m.p.) 63–64 °C; ^1^H-NMR (300 MHz, CDCl_3_): 4.02 (s, 2H, Cp), 4.05 (s, 5H, Cp), 4.19 (s, 2H, Cp), 4.83 (s, 1H, OH), 5.13 (s, 1H, CH), 6.76 (d, *J* = 8.1 Hz, 2H, Ar), 7.07 (d, *J* = 8.1 Hz, 2H, Ar), 7.19–7.33 (m, 5H, Ar); ^13^C-NMR (75 MHz, CDCl_3_): 51.0 (CH), 67.59 (CH), 67.63 (CH), 68.7 (CH), 92.0 (C), 114.9 (CH), 126.1 (CH), 128.1 (CH), 128.7 (CH), 129.9 (CH), 137.6 (C), 145.3 (C), 153.7 (C); HRMS (EI) calculated for [C_23_H_20_FeO]^+^ (M^+^): 368.0858, found 368.0856.

*4-[(Ferrocenyl)(2-methylphenyl)methyl]phenol* (**3b**): yellow solid; m.p. 66–67 °C; ^1^H-NMR (300 MHz, CDCl_3_): 2.25 (s, 3H, CH_3_), 3.81 (s, 1H, Cp), 4.07 (s, 5H, Cp), 4.16–4.12 (m, 1H, Cp), 4.20–4.16 (m, 1H, Cp), 4.76 (s, 1H, Cp), 5.25 (s, 1H, CH), 6.78 (d, *J* = 8.6 Hz, 2H, Ar), 6.96 (s, 1H, OH), 7.09–7.14 (m, 6H, Ar); ^13^C-NMR (75 MHz, CDCl_3_): 20.3 (CH_3_), 47.9 (CH), 67.4 (CH), 68.5 (CH), 68.8 (CH), 69.1 (CH), 69.9 (CH), 93.4 (C), 115.1 (CH), 125.9 (CH), 126.5 (CH), 128.6 (CH), 130.5 (CH), 130.9 (CH), 135.5 (C), 136.0 (C), 144.7 (C), 154.1 (C); HRMS (EI) calculated for [C_24_H_22_FeO]^+^ (M^+^): 382.1015, found 382.1009.

*4-[(Ferrocenyl)(3-methylphenyl)methyl]phenol* (**3c**): yellow solid; m.p. 72–73 °C; ^1^H-NMR (300 MHz, CDCl_3_): 2.32 (s, 3H, CH_3_), 4.02–4.05 (m, 7H, Cp), 4.18 (m, 2H, Cp), 4.62 (s, 1H, OH), 5.06 (s, 1H, CH), 6.75 (d, *J* = 8.4 Hz, 2H, Ar), 7.00–7.17 (m, 6H, Ar); ^13^C-NMR (75 MHz, CDCl_3_): 21.5 (CH_3_), 50.9 (CH), 67.7 (CH), 68.9 (CH), 92.3 (C), 114.7 (CH), 120.5 (CH), 125.6 (CH), 126.8 (CH), 127.9 (CH), 129.4 (CH), 129.9 (CH), 137.5 (C), 145.2 (C), 153.7 (C); HRMS (EI) calculated for [C_24_H_22_FeO]^+^ (M^+^): 382.1015, found 382.1011.

*4-[(Ferrocenyl)(4-methylphenyl)methyl]phenol* (**3d**): yellow solid; m.p. 64–65 °C; ^1^H-NMR (300 MHz, CDCl_3_): 2.33 (s, 3H, CH_3_), 3.99 (m, 2H, Cp), 4.03 (s, 5H, Cp), 4.17 (m, 2H, Cp), 4.73 (s, 1H, OH), 5.08 (s, 1H, CH), 6.74 (d, *J* = 8.5 Hz, 2H, Ar), 7.04–7.11 (m, 6H, Ar); ^13^C-NMR (75 MHz, CDCl_3_): 21.4 (CH_3_), 51.0 (CH), 67.9 (CH), 69.1 (CH), 92.6 (C), 115.2 (CH), 128.9 (CH), 129.1 (CH), 130.2 (CH), 135.9 (C), 138.2 (C), 142.7 (C), 154.1 (C); HRMS (EI) calculated for [C_24_H_22_FeO]^+^ (M^+^): 382.1015, found 382.1009.

*4-[(Ferrocenyl)(4-methoxyphenyl)methyl]phenol* (**3e**): yellow oil; ^1^H-NMR (300 MHz, CDCl_3_): 3.80 (s, 3H, OMe), 3.98 (s, 2H, Cp), 4.04 (s, 5H, Cp), 4.17 (s, 2H, Cp), 4.72 (s, 1H, CH), 5.06 (s, 1H, OH), 6.79–6.73 (m, 2H, Ar), 6.80–6.84 (m, 2H, Ar), 7.03–7.10 (m, 4H, Ar); ^13^C-NMR (75 MHz, CDCl_3_): 50.5 (CH), 55.7 (CH_3_), 68.0 (CH), 69.2 (CH), 92.9 (C), 113.8 (CH), 115.2 (CH), 130.0 (CH), 130.2 (CH), 138.1 (C), 138.3 (C), 154.1 (C), 158.2 (C); HRMS (EI) calculated for [C_24_H_22_FeO_2_]^+^ (M^+^): 398.0964, found 398.0972.

*4-(1-Ferrocenylethyl)phenol* (**3f**): yellow solid; m.p. = 95–96 °C; ^1^H-NMR (300 MHz, CDCl_3_): 1.57 (d, *J* = 7.2 Hz, 3H, CH_3_), 3.90 (q, *J* = 7.2 Hz, 1H, CH), 4.09 (s, 1H, Cp), 4.10–4.18 (m, 8H, Cp), 4.63 (s, 1H, OH), 6.74 (d, *J* = 8.6 Hz, 2H, Ar), 7.05 (d, *J* = 8.6 Hz, 2H, Ar); ^13^C-NMR (75 MHz, CDCl_3_): 23.1 (CH_3_), 39.3 (CH), 66.7 (CH), 67.3 (CH), 67.9 (CH), 68.2 (CH), 69.0 (CH), 95.1 (C), 115.4 (CH), 128.6 (CH), 140.4 (C), 153.9 (C); HRMS (EI) calculated for [C_18_H_18_FeO]^+^ (M^+^): 306.0702, found 306.0701. Ferrocene **3f** is a known compound; our characterization data match those previously reported in the literature [25].

*4-(1-Ferrocenylpropyl)phenol* (**3g**): yellow solid; m.p. = 87–88 °C; ^1^H-NMR (300 MHz, CDCl_3_): 0.85 (t, *J* = 7.4 Hz, 3H, CH_3_), 1.67–1.83 (m, 1H, CH_2_), 2.07–2.14 (m, 1H, CH_2_), 3.41–3.46 (dd, *J* = 10.7 and 4.3 Hz, 1H, CH), 4.06 (s, 1H, Cp), 4.05–4.10 (m, 7H, Cp), 4.17 (s, 1H, Cp), 4.73 (s, 1H, OH), 6.78 (d, *J* = 8.5 Hz, 2H, Ar), 7.06 (d, *J* = 8.5 Hz, 2H, Ar); ^13^C-NMR (75 MHz, CDCl_3_): 12.7 (CH_3_), 30.0 (CH_2_), 47.0 (CH), 66.8 (CH), 66.9 (CH), 67.3 (CH), 67.4 (CH), 68.6 (CH), 94.8 (C), 114.9 (CH), 129.0 (CH), 137.8 (C), 153.6 (C); HRMS (EI) calculated for [C_19_H_20_FeO]^+^ (M^+^): 320.0858, found 320.0853.

*4-(1-Ferrocenylpentyl)phenol* (**3h**): yellow oil; ^1^H-NMR (300 MHz, CDCl_3_): 0.89 (t, *J* = 7.0 Hz, 3H, CH_3_), 1.16–1.40 (m, 4H, CH_2_), 1.70–1.82 (m, 1H, CH_2_), 2.01–2.08 (m, 1H, CH_2_), 3.53 (dd, *J* = 10.8 and 4.3 Hz, 1H, CH), 3.95 (s, 1H, Cp), 4.05–4.11 (m, 7H, Cp), 4.18 (s, 1H, Cp), 4.80 (s, 1H, OH), 6.76 (d, *J* = 8.3 Hz, 2H, Ar), 7.06 (d, *J* = 8.3 Hz, 2H, Ar); ^13^C-NMR (75 MHz, CDCl_3_): 14.1 (CH_3_), 22.7 (CH_2_), 30.2 (CH_2_), 36.8 (CH_2_), 45.1 (CH), 66.8 (CH), 66.9 (CH), 67.3 (CH), 67.4 (CH), 68.6 (CH), 95.1 (C), 114.9 (CH), 128.9 (CH), 138.1 (C), 153.6 (C); HRMS (EI) calculated for [C_21_H_24_FeO]^+^ (M^+^): 348.1171, found 348.1184.

*4-[1-(Ferrocenyl)(2-methyl)propyl]phenol* (**3i**): yellow solid; m.p. = 95–96 °C; ^1^H-NMR (300 MHz, CDCl_3_): 0.74 (d, *J* = 6.6 Hz, 3H, CH_3_), 0.92 (d, *J* = 6.6 Hz, 3H, CH_3_), 1.97–2.03 (m, 1H, CH), 3.08 (d, *J* = 8.7 Hz, 1H, CH), 3.94 (s, 5H, Cp), 4.13–4.27 (m, 4H, Cp), 4.74 (s, 1H, OH), 6.75 (d, *J* = 8.3 Hz, 2H, Ar), 7.05 (d, *J* = 8.3 Hz, 2H, Ar); ^13^C-NMR (75 MHz, CDCl_3_): 21.7 (CH_3_), 22.2 (CH_3_), 53.1 (CH), 66.7 (CH), 67.2 (CH), 68.4 (CH), 69.3 (CH), 70.4 (CH), 94.7 (C), 114.9 (CH), 129.6 (CH), 137.8 (C), 153.5 (C); HRMS (EI) calculated for [C_20_H_22_FeO]^+^ (M^+^): 334.1015, found 334.1012.

*4-[1-(Ferrocenyl)(2,2-dimethyl)propyl]phenol* (**3j**): yellow solid; m.p. = 105–106 °C; ^1^H-NMR (300 MHz, CDCl_3_): 0.84 (s, 9H, CH_3_), 3.23 (s, 1H, CH), 3.73 (s, 5H, Cp), 4.07 (s, 1H, Cp), 4.14 (d, *J* = 6.2 Hz, 2H, Cp), 4.24 (s, 1H, Cp), 4.93 (s, 1H, OH), 6.84 (d, *J* = 8.4 Hz, 2H, Ar), 7.27–7.29 (m, 2H, Ar); ^13^C-NMR (75 MHz, CDCl_3_): 28.7 (CH_3_), 35.2 (C), 57.3 (CH), 65.7 (CH), 68.0 (CH), 68.1 (CH), 68.5 (CH), 69.2 (CH), 72.1 (CH), 90.4 (C), 114.2 (CH), 132.0 (CH), 136.9 (C), 153.6 (C); HRMS (EI) calculated for [C_21_H_24_FeO]^+^ (M^+^): 348.1171, found 348.1182.

*4-[1-(Ferrocenyl)but-3-enyl]phenol* (**3k**): yellow oil; ^1^H-NMR (300 MHz, CDCl_3_): 2.53–2.62 (m, 1H, CH_2_), 2.80–2.89 (m, 1H, CH_2_), 3.65 (dd, *J* = 10.5 and 4.7 Hz, 1H, CH), 4.08 (s, 1H, Cp), 4.13–4.10 (m, 7H, Cp), 4.19 (s, 1H, Cp), 4.67 (s, 1H, OH), 4.93–5.04 (m, 2H, =CH_2_), 5.76–5.77 (m, 1H, =CH), 6.76 (d, *J* = 8.6 Hz, 2H, Ar), 7.06 (d, *J* = 8.6 Hz, 2H, Ar); ^13^C-NMR (75 MHz, CDCl_3_): 12.7 (CH_3_), 30.0 (CH_2_), 47.0 (CH), 66.8 (CH), 66.9 (CH), 67.3 (CH), 67.4 (CH), 68.6 (CH), 94.8 (C), 114.9 (CH), 129.0 (CH), 137.8 (C), 153.6 (C); HRMS (EI) calculated for [C_20_H_20_FeO]^+^ (M^+^): 332.0858, found 332.0855.

*4-(Ferrocenylmethyl)phenol* (**3l**): yellow oil; ^1^H-NMR (300 MHz, CDCl_3_): 3.64 (s, 2H, CH_2_), 4.11 (s, 4H, Cp), 4.15 (s, 5H, Cp), 4.84 (s, 1H, OH), 6.75 (d, *J* = 8.2 Hz, 2H, Ar), 7.02 (d, *J* = 8.2 Hz, 2H, Ar); ^13^C-NMR (75 MHz, CDCl_3_): 45.2 (CH_2_), 77.6 (CH), 78.7 (CH), 78.8 (CH), 98.7 (C), 125.1 (CH), 139.6 (CH), 144.0 (C), 163.7 (C); HRMS (EI) calculated for [C_17_H_16_FeO]^+^ (M^+^): 292.0545, found 292.0524. Ferrocene **3l** is a known compound [25].

### 3.3. Synthesis of Ferrocene Derivative ***5***

InCl_3_ (4.4 mg, 0.02 mmol, 10 mol%) was added to a solution of 4-benzylidene-2,6-di-*tert*-butylcyclohexa-2,5-dienone **4** (58.9 mg, 0.2 mmol) and ferrocene **2** (111.6 mg, 0.6 mmol) in toluene (2 mL). The mixture was stirred at 100 °C for 6 h (disappearance of **4** checked by TLC). Then, the solvent was removed under reduced pressure and the resulting residue was purified by flash chromatography (silica gel, hexanes/ethyl acetate 5:1) to yield ferrocene derivative **5** (76.9 mg, 80%) as a yellow oil; ^1^H-NMR (300 MHz, CDCl_3_): 1.44 (s, 18H, CH_3_), 3.98–3.99 (m, 1H, Cp), 4.01 (s, 5H, Cp), 4.03–4.04 (m, 1H, Cp), 4.16–4.17 (m, 1H, Cp), 4.91 (s, 1H, Cp), 5.06 (s, 1H, CH), 5.09 (s, 1H, OH), 7.04 (s, 2H, Ar), 7.18–7.21 (m, 3H, Ar), 7.26–7.29 (m, 2H, Ar); ^13^C-NMR (75 MHz, CDCl_3_): 30.0 (CH_3_), 34.4 (CH), 51.8 (CH), 67.3 (CH), 67.6 (CH), 68.6 (CH), 68.7 (CH), 68.8 (CH), 92.8 (C), 125.4 (CH), 125.8 (C), 127.9 (CH), 128.6 (CH), 135.1 (C), 135.4 (C), 143.8 (C), 151.9 (C); HRMS (EI) calculated for [C_31_H_36_FeO]^+^ (M^+^): 480.2110, found 480.2124.

### 3.4. Cytotoxic Assays

Cell Counting Kit-8 (CCK-8) from Sigma-Aldrich (Madrid, Spain) was used according to the protocol provided by the company. The A2780 and A549 cell lines were used in this preliminary study. First, cell lines were cultured for 7 days in Dulbecco’s modified Eagle’s medium (DMEM) supplemented with 10% (*v*/*v*) fetal bovine serum (FBS). Then, cells were seeded into a 96-well flat-bottom culture plate at a cell density of 500–2000 cells/well and incubated for 24 h in the same medium (DMEM/10% FBS). After that, 10 μL of a solution of the corresponding ferrocene derivative at different concentrations were added and the cells were incubated for 72 h. Then, 10 μL of the CCK-8 solution were added to each well of the plate. After 2 h of incubation the absorbance at 450 nm was recorded using a BioTek ELx800 Absorbance Microplate Reader (BioTek, Bad Friedrichshall, Germany). Measurements were performed in triplicate, and each experiment was repeated three times. The IC_50_ values (μm) were estimated by treatment of the data obtained with the statistical program GraphPad Prism5 (version 5.04).

## 4. Conclusions

Guided by earlier work from our group, we have developed a convenient synthesis of *para*-substituted phenol derivatives containing a ferrocenyl moiety. Salient features of our protocol include (i) easy availability of the required starting materials, (ii) synthetically useful yields, and (iii) mild reaction conditions. This C–H bond functionalization of ferrocene relies on the generation of a *para*-quinone methide intermediate that, activated by Lewis acid complexation, would serve as electrophilic partner in an aromatic electrophilic substitution. Preliminary biological evaluation revealed that some of the ferrocene derivatives available by this protocol display significant cytotoxicity against ovarian and lung cancer cell lines. Further studies aimed at the preparation of new ferrocene derivatives with enhanced antiproliferative properties are being pursued in our laboratory.

## Figures and Tables

**Table 1:**
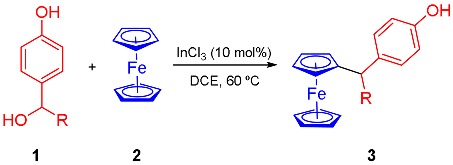
InCl_3_-catalyzed reaction of *p*-hydroxybenzyl alcohols **1** and ferrocene (**2**).

Entry	Substrate	R	3	Yield (%) ^a^
1	**1a**	C_6_H_5_	**3a**	75
2	**1b**	*o*-MeC_6_H_4_	**3b**	76
3	**1c**	*m*-MeC_6_H_4_	**3c**	51
4	**1d**	*p*-MeC_6_H_4_	**3d**	82
5	**1e**	*p*-MeOC_6_H_4_	**3e**	48
6	**1f**	Me	**3f**	62
7	**1g**	Et	**3g**	60
8	**1h**	*n*-Bu	**3h**	42
9	**1i**	*i*-Pr	**3i**	62
10	**1j**	*t*-Bu	**3j**	43
11	**1k**	Allyl	**3k**	44
12	**1l**	H	**3l**	64

^a^ Isolated yield after chromatographic purification.

**Table 2:** IC_50_ [μM] values for selected ferrocenyl compounds on different cell lines ^a^.

	3a	3g
**A2780**	1.07	2.23
**A549**	3.55	4.89

^a^ Measured after 72 h of culture.

**Figure A1:**
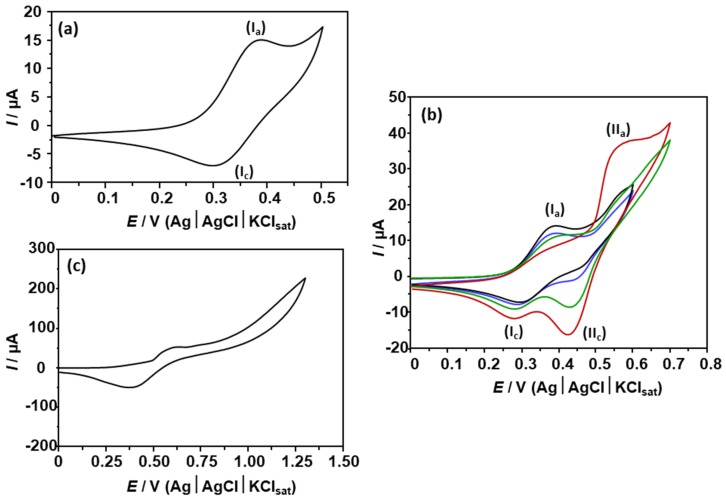
Cyclic voltammetry of compound **3a** incorporated to the carbon paste electrode in phosphate saline buffer (pH 7.4). Scan rate = 50 mV/s. Potential scan from 0 V to +0.5 V; (**a**) +0.6 (first scan, black; second scan, blue) and +0.7 (first scan, green; second scan, red) (**b**) and +1.3 V (**c**).

## Supplementary Materials

Supplementary Materials available online: Copies of ^1^H- and ^13^C-NMR spectra, X-Ray crystallography (printcif and checkcif).

## Appendix A. Crystal Data for Ferrocene Derivative 3a

Crystal Data for C_23_H_20_FeO (*M*_r_ = 368.24 g/mol): monoclinic, space group *P*2_1_/n (No. 14), *a* = 15.662(1) Å, *b* = 6.0219(3) Å, *c* = 18.786(1) Å, *β* = 96.990(6)°, *V* = 1758.6(2) Å^3^, *Z* = 4, *T* = 299 K, *μ*(Cu*Kα*) = 6.91 mm^−1^, D_x_ = 1.391 g/cm^3^, 8336 measured reflections (3.5° < *θ* < 69.6°), 3254 independent reflections, 2641 observed reflections (*I* > 2*σ*(*I*)), *R*_int_ = 0.045. Final *R*[*F*^2^ > 2*σ*(*F*^2^)] was 0.079 and *wR*(*F*^2^) = 0.255.

## Appendix B. Electrochemical Study for Ferrocene Derivative 3a

Compound **3a** was studied by cyclic voltammetry (CV). All potentials were referred to a Ag|AgCl|KCl_(sat)_ reference electrode. The ferrocene/ferrocenium couple (I_a_/I_c_) is clearly observed at a formal potential, E°′ = 0.352 V (Figure A1a). The anodic peak current is higher than the cathodic one indicating that some ferrocenium ions diffuse from the carbon paste electrode to the bulk solution due to the positive charge of the cation. Nucleation at potentials ~0.450 V points out to a dissimilar electrochemical behavior with other structurally related compounds. When the potential is swept up to +0.6 V, a second redox process (II_a_/II_c_) appears causing the decrease of I_a_ (Figure A1b). The reduction of ferrocenium species (I_c_) remains visible but progressively shifted towards less positive potentials in subsequent scans. Process II_a_ at about +0.570 V is clear and well-shaped but process II_c_ at 0.426 V is partially overlapped with I_c_. Of note, a notably increase in the non-faradaic current is observed. When the potential is extended to +1.3 V, additional oxidation reactions take place (see the rising anodic current) probably associated to phenolic compounds. Only one reduction process is observed as a result of I_c_ and II_c_ overlapping (Figure A1c). The potential of both oxidation and reduction peaks shift to more extreme potentials which indicates that the process is irreversible. The origin of the irreversibility might be the formation of non-conducting products on the electrode surface that hinder the electron transfer. The appearance of resistance affecting the CV shape strongly supports this explanation.
