# Supplementary material for: Trapping para-Quinone Methide Intermediates with Ferrocene: Synthesis and Preliminary Biological Evaluation of New Phenol-Ferrocene Conjugates
Source: Molecules. 2018 Jun 1;23(6):1335. doi: 10.3390/molecules23061335 (PMC6099632; doi:10.3390/molecules23061335)

checkCIF/PLATON report

Structure factors have been supplied for datablock(s) 3a

THIS REPORT IS FOR GUIDANCE ONLY. IF USED AS PART OF A REVIEW PROCEDURE FOR PUBLICATION, IT SHOULD NOT REPLACE THE EXPERTISE OF AN EXPERIENCED CRYSTALLOGRAPHIC REFEREE.

No syntax errors found.   CIF dictionary   Interpreting this report

Datablock: 3a

```
Bond precision:  C-C = 0.0108 A          Wavelength=1.54184

Cell:           a=15.6620(12)    b=6.0219(3)       c=18.7859(11)
                alpha=90         beta=96.990(6)    gamma=90
Temperature:    299 K

                Calculated                Reported
Volume          1758.62(19)               1758.6(2)
Space group     P 21/n                    P 21/n
Hall group      -P 2yn                    -P 2yn
Moiety formula  C23 H20 Fe O              ?
Sum formula     C23 H20 Fe O              C23 H20 Fe O
Mr              368.24                    368.24
Dx,g cm-3       1.391                     1.391
Z               4                         4
Mu (mm-1)       6.911                     6.911
F000            768.0                     768.0
F000'           765.33
h,k,lmax        19,7,22                   18,7,22
Nref            3307                      3254
Tmin,Tmax       0.498,0.871               0.675,1.000
Tmin'           0.120

Correction method= # Reported T Limits: Tmin=0.675 Tmax=1.000
AbsCorr = MULTI-SCAN

Data completeness= 0.984          Theta(max)= 69.596

R(reflections)= 0.0789( 2641)     wR2(reflections)= 0.2554( 3254)

S = 1.118                  Npar= 238
```

The following ALERTS were generated. Each ALERT has the format
test-name_ALERT_alert-type_alert-level.
Click on the hyperlinks for more details of the test.

Alert level B

```
PLAT420_ALERT_2_B D-H Without Acceptor       O4       --H4O            Please Check
PLAT930_ALERT_2_B Check Twin Law ( 1 0  1  )[ 4 0  3  ] Estimated BASF       0.28
```

Alert level C

```
PLAT084_ALERT_3_C High wR2 Value (i.e. > 0.25) ...................       0.26 Report
PLAT241_ALERT_2_C High   'MainMol' Ueq as Compared to Neighbors of        C14 Check
PLAT242_ALERT_2_C Low    'MainMol' Ueq as Compared to Neighbors of        Fe1 Check
PLAT341_ALERT_3_C Low Bond Precision on  C-C Bonds ...............    0.01076 Ang.
PLAT906_ALERT_3_C Large K Value in the Analysis of Variance ......      3.633 Check
PLAT906_ALERT_3_C Large K Value in the Analysis of Variance ......      2.054 Check
PLAT911_ALERT_3_C Missing FCF Refl Between Thmin & STh/L=    0.600          5 Report
PLAT934_ALERT_3_C Number of (Iobs-Icalc)/SigmaW > 10 Outliers ....          1 Check
```

Alert level G

```
PLAT007_ALERT_5_G Number of Unrefined Donor-H Atoms ..............          2 Report
PLAT072_ALERT_2_G SHELXL First  Parameter in WGHT  Unusually Large       0.12 Report
PLAT301_ALERT_3_G Main Residue  Disorder ..............(Resd  1  )         4% Note
PLAT793_ALERT_4_G Model has Chirality at C7          (Centro SPGR)          R Verify
PLAT794_ALERT_5_G Tentative Bond Valency for Fe1       (II)      .       2.13 Info
PLAT870_ALERT_4_G ALERTS Related to Twinning Effects Suppressed ..          ! Info
PLAT912_ALERT_4_G Missing # of FCF Reflections Above STh/L=  0.600         47 Note
PLAT931_ALERT_5_G Found Twin Law ( 1 0  1  )[         ] Est.  BASF       0.28 Check
```

---

0 **ALERT level A** = Most likely a serious problem - resolve or explain
2 **ALERT level B** = A potentially serious problem, consider carefully
8 **ALERT level C** = Check. Ensure it is not caused by an omission or oversight
8 **ALERT level G** = General information/check it is not something unexpected

0 ALERT type 1 CIF construction/syntax error, inconsistent or missing data
5 ALERT type 2 Indicator that the structure model may be wrong or deficient
7 ALERT type 3 Indicator that the structure quality may be low
3 ALERT type 4 Improvement, methodology, query or suggestion
3 ALERT type 5 Informative message, check

It is advisable to attempt to resolve as many as possible of the alerts in all categories. Often the minor alerts point to easily fixed oversights, errors and omissions in your CIF or refinement strategy, so attention to these fine details can be worthwhile. In order to resolve some of the more serious problems it may be necessary to carry out additional measurements or structure refinements. However, the purpose of your study may justify the reported deviations and the more serious of these should normally be commented upon in the discussion or experimental section of a paper or in the "special_details" fields of the CIF. checkCIF was carefully designed to identify outliers and unusual parameters, but every test has its limitations and alerts that are not important in a particular case may appear. Conversely, the absence of alerts does not guarantee there are no aspects of the results needing attention. It is up to the individual to critically assess their own results and, if necessary, seek expert advice.

Publication of your CIF in IUCr journals

A basic structural check has been run on your CIF. These basic checks will be run on all CIFs submitted for publication in IUCr journals (*Acta Crystallographica*, *Journal of Applied Crystallography*, *Journal of Synchrotron Radiation*); however, if you intend to submit to *Acta Crystallographica Section C* or *E* or *IUCrData*, you should make sure that full publication checks are run on the final version of your CIF prior to submission.

Publication of your CIF in other journals

Please refer to the *Notes for Authors* of the relevant journal for any special instructions relating to CIF submission.

PLATON version of 23/04/2018; check.def file version of 23/04/2018

Datablock 3a - ellipsoid plot

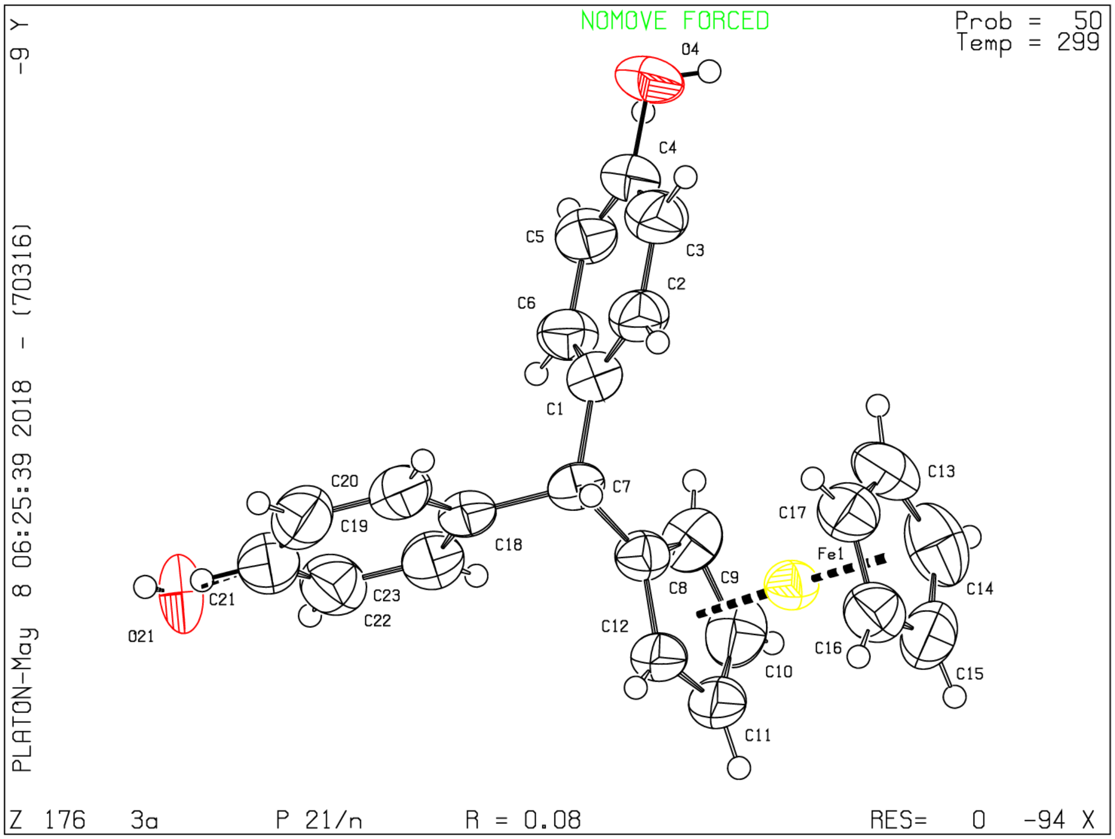

Supplement: Supplementary file 1 [file molecules-23-01335-s001.zip › Supplementary Files/checkcif-1.pdf]
