# Supplementary material for: Trapping para-Quinone Methide Intermediates with Ferrocene: Synthesis and Preliminary Biological Evaluation of New Phenol-Ferrocene Conjugates
Source: Molecules. 2018 Jun 1;23(6):1335. doi: 10.3390/molecules23061335 (PMC6099632; doi:10.3390/molecules23061335)
Supplement: Supplementary file 1 [file molecules-23-01335-s001.zip › Supplementary Files/printcif.pdf]

Trapping *para*-Quinone Methide Intermediates with Ferrocene: Synthesis and Preliminary Biological Evaluation of New Phenol-Ferrocene Conjugates

Silvia González-Pelayo,[a] Enol López H.,[a] Javier Borge,[b] Noemí de-los-Santos-Álvarez[c] and Luis A. López[a]*

[a]Departamento de Química Orgánica e Inorgánica, Instituto Universitario de Química Organometálica "Enrique Moles", Universidad de Oviedo, Julián Clavería 8, 33006 Oviedo, Spain, [b]Departamento de Química Física y Analítica, Centro de Innovación en Química Avanzada (ORFEO-CINQA), Universidad de Oviedo, Julián Clavería 8, 33006 Oviedo, Spain, and [c]Departamento de Química Física y Analítica, Universidad de Oviedo, Julián Clavería 8, 33006 Oviedo, Spain

Correspondence email: lalg@uniovi.es

Abstract

The reaction of *para*-hydroxybenzyl alcohols with ferrocene in the presence of a catalytic amount of $InCl_3$ provided ferrocenyl phenol derivatives, an interesting class of organometallic compounds with potential applications in medicinal chemistry. This transformation exhibited a reasonable substrate scope delivering the desired products in synthetically useful yields. Evidence of involvement of a *para*-quinone methide intermediate in this coupling process was also provided. Preliminary biological evaluation demonstrated that some of the ferrocene derivatives available by this methodology exhibit significant cytotoxicity against several cancer cell lines with IC_{50} values withing the range of 1.07–4.89 μM.

1. Synthesis and crystallization

Crystals of compound **'3a'**, suitable for X-ray diffraction analysis, were obtained from diffusion of pentane into dichlorometane solutions at −20 °C. Data collection was performed with a Rigaku-Oxford Diffraction Xcalibur Onyx Nova single-crystal diffractometer using Cu–*Kα* radiation (λ = 1.5418 Å). Images were collected at a fixed crystal-detector distance of 62 mm, using the oscillation method with 1.30° oscillation and 20.0–215.0 s variable exposure time per image. Data collection strategy was calculated with the program *CrysAlis^Pro*[1]. Data reduction and cell refinement were also performed with the program *CrysAlis^Pro*[1]. An empirical absorption correction was applied using the SCALE3 ABSPACK algorithm as implemented in the program *CrysAlis^Pro*[1]. The most relevant crystal and refinement data are collected in Table 1.

2. Refinement

The structure was solved by Patterson interpretation and phase expansion using *DIRDIF2008*[2]. Isotropic least-squares refinements on F^2 using *SHELXL2014*[3] were performed. During the final stages of the refinements, all the positional and anisotropic displacement parameters of all non-H atoms were refined. The H atoms were geometrically located and their coordinates were refined riding on their parent atoms. The crystallographic plots were made with *PLATON*[4] and *Mercury CSD 3.8*[5].

Table 1

Experimental details

Crystal data	
Chemical formula	$C_{23}H_{20}FeO$
M_r	368.24
Crystal system, space group	Monoclinic, $P2_1/n$
Temperature (K)	299

a, b, c (Å)	15.6620 (12), 6.0219 (3), 18.7859 (11)
β (°)	96.990 (6)
V (Å^3)	1758.6 (2)
Z	4
Radiation type	Cu $K\alpha$
μ (mm^{-1})	6.91
Crystal size (mm)	0.30 × 0.09 × 0.02
Data collection	
Diffractometer	Xcalibur, Onyx, Nova
Absorption correction	Multi-scan *CrysAlis PRO* 1.171.38.43 (Rigaku Oxford Diffraction, 2015). Empirical absorption correction using spherical harmonics, implemented in SCALE3 ABSPACK scaling algorithm.
T_{min}, T_{max}	0.675, 1.000
No. of measured, independent and observed [$I > 2\sigma(I)$] reflections	8336, 3254, 2641
R_{int}	0.045
(sin $\theta/\lambda)_{max}$ (Å^{-1})	0.608
Refinement	
$R[F^2 > 2\sigma(F^2)]$, $wR(F^2)$, S	0.079, 0.255, 1.12
No. of reflections	3254
No. of parameters	238
H-atom treatment	H-atom parameters constrained
$\Delta\rho_{max}$, $\Delta\rho_{min}$ (e Å^{-3})	0.95, −0.72

Computer programs: *CrysAlis^{Pro}* (Rigaku Oxford Diffraction, 2015), *DIRDIF2008* (Beurskens *et al.*, 2008), *SHELXL2014* (Sheldrick, 2015), *PLATON* (Spek, 2009) / *Mercury* CSD 3.8 (Macrae *et al.*, 2008).

Acknowledgements

Funding information

References

[1] Rigaku Oxford Diffraction. **2015**. *CrysAlis^{Pro}*.

[2] Beurskens, P. T.; Beurskens, G.; de Gelder, R.; García-Granda, S.; Gould, R. O.; Smits, J. M. M. **2008**. *The DIRDIF2008 program system*. Crystallography Laboratory, University of Nijmegen: Nijmegen, The Netherlands.

[3] Sheldrick, G. M. *Acta Cryst.* **2015**, C71, 3-8.

[4] Spek, A. L. *Acta Cryst.* **2009**, D65, 148-155.

[5] Macrae, C. F.; Bruno, I. J.; Chisholm, J. A.; Edgington, P. R.; McCabe, P.; Pidcock, E.; Rodríguez-Monge, L.; Taylor, R.; van de Streek, J.; Wood, P. A. *J. Appl. Cryst.* **2008**, 41, 466-470.

Figure 1

supporting information

Trapping *para*-Quinone Methide Intermediates with Ferrocene: Synthesis and Preliminary Biological Evaluation of New Phenol-Ferrocene Conjugates

Computing details

Data collection: *CrysAlis^{Pro}* (Rigaku Oxford Diffraction, 2015); cell refinement: *CrysAlis^{Pro}* (Rigaku Oxford Diffraction, 2015); data reduction: *CrysAlis^{Pro}* (Rigaku Oxford Diffraction, 2015); program(s) used to solve structure: *DIRDIF2008* (Beurskens *et al.*, 2008); program(s) used to refine structure: *SHELXL2014* (Sheldrick, 2015); molecular graphics: *PLATON* (Spek, 2009) / *Mercury* CSD 3.8 (Macrae *et al.*, 2008).

(3a)

Crystal data

$C_{23}H_{20}FeO$
M_r = 368.24
Monoclinic, $P2_1/n$
a = 15.6620 (12) Å
b = 6.0219 (3) Å
c = 18.7859 (11) Å
β = 96.990 (6)°
V = 1758.6 (2) Å^3
Z = 4
$F(000)$ = 768
D_x = 1.391 Mg m^{-3}
Cu $K\alpha$ radiation, λ = 1.54184 Å
Cell parameters from 2708 reflections
θ = 3.5–68.3°
μ = 6.91 mm^{-1}
T = 299 K
Prism, orange
0.30 × 0.09 × 0.02 mm

Data collection

Xcalibur, Onyx, Nova diffractometer
Radiation source: micro-focus sealed X-ray tube, Nova (Cu) X-ray Source
Detector resolution: 8.2640 pixels mm^{-1}
Absorption correction: multi-scan *CrysAlis PRO* 1.171.38.43 (Rigaku Oxford Diffraction, 2015). Empirical absorption correction using spherical harmonics, implemented in SCALE3 ABSPACK scaling algorithm.
T_{min} = 0.675, T_{max} = 1.000
8336 measured reflections
3254 independent reflections
2641 reflections with $I > 2\sigma(I)$
R_{int} = 0.045
θ_{max} = 69.6°, θ_{min} = 3.5°
h = −18→18
k = −4→7
l = −21→22

Refinement

Refinement on F^2
Least-squares matrix: full
$R[F^2 > 2\sigma(F^2)]$ = 0.079
$wR(F^2)$ = 0.255
S = 1.12
3254 reflections
238 parameters
0 restraints
Hydrogen site location: inferred from neighbouring sites
H-atom parameters constrained
$w = 1/[\sigma^2(F_o^2) + (0.1187P)^2 + 4.0893P]$ where $P = (F_o^2 + 2F_c^2)/3$
$(\Delta/\sigma)_{max}$ < 0.001
$\Delta\rho_{max}$ = 0.95 e Å^{-3}
$\Delta\rho_{min}$ = −0.72 e Å^{-3}

Special details

Geometry. All e.s.d.'s (except the e.s.d. in the dihedral angle between two l.s. planes) are estimated using the full covariance matrix. The cell e.s.d.'s are taken into account individually in the estimation of e.s.d.'s in distances, angles and torsion angles; correlations between e.s.d.'s in cell parameters are only used when they are defined by crystal symmetry. An approximate (isotropic) treatment of cell e.s.d.'s is used for estimating e.s.d.'s involving l.s. planes.

Fractional atomic coordinates and isotropic or equivalent isotropic displacement parameters (Å^2)

	x	y	z	U_{iso}*/U_{eq}	Occ. (<1)
Fe1	0.08144 (6)	0.17743 (15)	0.35123 (5)	0.0530 (4)	
C8	0.2121 (4)	0.2094 (10)	0.3784 (3)	0.0529 (13)	
C9	0.1844 (5)	−0.0096 (11)	0.3924 (4)	0.0677 (17)	
H9	0.1904	−0.0808	0.4367	0.081*	
C10	0.1456 (5)	−0.0998 (13)	0.3259 (5)	0.078 (2)	
H10	0.1227	−0.2420	0.3195	0.094*	
C11	0.1473 (4)	0.0589 (14)	0.2722 (4)	0.072 (2)	
H11	0.1254	0.0416	0.2243	0.087*	
C12	0.1884 (4)	0.2515 (13)	0.3034 (3)	0.0612 (15)	
H12	0.1982	0.3824	0.2794	0.073*	
C13	0.0022 (6)	0.2293 (15)	0.4283 (4)	0.081 (2)	
H13	0.0153	0.1961	0.4767	0.097*	
C14	−0.0375 (6)	0.0865 (15)	0.3758 (6)	0.094 (3)	
H14	−0.0564	−0.0570	0.3833	0.113*	
C15	−0.0438 (5)	0.2001 (16)	0.3082 (5)	0.087 (3)	
H15	−0.0665	0.1439	0.2638	0.104*	
C16	−0.0090 (5)	0.4139 (14)	0.3224 (4)	0.0723 (18)	
H16	−0.0051	0.5250	0.2885	0.087*	
C17	0.0187 (5)	0.4311 (13)	0.3956 (4)	0.0716 (18)	
H17	0.0439	0.5555	0.4188	0.086*	
C7	0.2640 (4)	0.3660 (9)	0.4282 (3)	0.0496 (12)	
H7	0.2404	0.5147	0.4175	0.059*	
C1	0.2573 (4)	0.3236 (10)	0.5076 (3)	0.0548 (14)	
C2	0.2125 (4)	0.4666 (13)	0.5465 (3)	0.0653 (16)	
H2	0.1861	0.5902	0.5237	0.078*	
C3	0.2054 (5)	0.4325 (17)	0.6181 (4)	0.083 (2)	
H3	0.1756	0.5332	0.6433	0.099*	
C4	0.2428 (5)	0.2488 (18)	0.6514 (4)	0.082 (2)	
H4	0.2374	0.2234	0.6995	0.098*	0.205 (15)
O4	0.2336 (5)	0.226 (2)	0.7224 (3)	0.114 (4)	0.795 (15)
H4O	0.1843	0.1871	0.7266	0.171*	0.795 (15)
C5	0.2881 (5)	0.1016 (14)	0.6147 (4)	0.0735 (19)	
H5	0.3133	−0.0233	0.6376	0.088*	
C6	0.2957 (4)	0.1411 (12)	0.5434 (3)	0.0625 (16)	
H6	0.3274	0.0431	0.5187	0.075*	
C18	0.3575 (4)	0.3722 (10)	0.4130 (3)	0.0525 (13)	
C19	0.4045 (5)	0.5675 (12)	0.4272 (3)	0.0674 (17)	
H19	0.3786	0.6891	0.4464	0.081*	
C20	0.4883 (5)	0.5823 (16)	0.4132 (4)	0.081 (2)	
H20	0.5188	0.7138	0.4226	0.097*	
C21	0.5269 (5)	0.4042 (17)	0.3855 (4)	0.084 (2)	
H21	0.5838	0.4142	0.3761	0.101*	0.795 (15)
O21	0.6030 (15)	0.387 (6)	0.3677 (19)	0.097 (11)	0.205 (15)

H21O	0.6269	0.5087	0.3717	0.146*	0.205 (15)
C22	0.4819 (5)	0.2097 (15)	0.3715 (4)	0.081 (2)	
H22	0.5085	0.0883	0.3530	0.097*	
C23	0.3968 (5)	0.1942 (11)	0.3849 (4)	0.0665 (17)	
H23	0.3664	0.0629	0.3749	0.080*	

Atomic displacement parameters (Å^2)

	U^{11}	U^{22}	U^{33}	U^{12}	U^{13}	U^{23}
Fe1	0.0569 (6)	0.0544 (6)	0.0479 (5)	0.0002 (4)	0.0079 (4)	−0.0052 (4)
C8	0.057 (3)	0.052 (3)	0.049 (3)	0.003 (3)	0.007 (2)	−0.003 (2)
C9	0.072 (4)	0.050 (3)	0.078 (4)	0.000 (3)	−0.001 (3)	−0.006 (3)
C10	0.070 (4)	0.063 (4)	0.100 (6)	0.002 (3)	0.006 (4)	−0.035 (4)
C11	0.062 (4)	0.100 (6)	0.055 (3)	0.000 (4)	0.008 (3)	−0.029 (4)
C12	0.060 (3)	0.078 (4)	0.046 (3)	0.002 (3)	0.007 (3)	−0.009 (3)
C13	0.088 (5)	0.096 (6)	0.066 (4)	0.015 (5)	0.033 (4)	0.012 (4)
C14	0.086 (5)	0.067 (5)	0.137 (8)	−0.006 (4)	0.044 (6)	0.003 (5)
C15	0.058 (4)	0.109 (7)	0.091 (6)	0.002 (4)	−0.001 (4)	−0.031 (5)
C16	0.071 (4)	0.078 (5)	0.068 (4)	0.012 (4)	0.012 (3)	0.012 (4)
C17	0.073 (4)	0.073 (4)	0.069 (4)	0.007 (4)	0.013 (3)	−0.012 (4)
C7	0.061 (3)	0.043 (3)	0.043 (3)	0.002 (2)	0.001 (2)	−0.001 (2)
C1	0.058 (3)	0.057 (3)	0.048 (3)	0.000 (3)	0.002 (3)	−0.001 (3)
C2	0.064 (4)	0.079 (4)	0.052 (3)	0.012 (3)	0.004 (3)	−0.011 (3)
C3	0.070 (4)	0.122 (7)	0.057 (4)	0.017 (5)	0.011 (3)	−0.016 (4)
C4	0.068 (4)	0.136 (7)	0.043 (3)	−0.004 (5)	0.011 (3)	0.001 (4)
O4	0.087 (5)	0.216 (11)	0.041 (3)	0.005 (6)	0.017 (3)	0.016 (5)
C5	0.071 (4)	0.093 (5)	0.056 (4)	0.005 (4)	0.003 (3)	0.019 (4)
C6	0.069 (4)	0.069 (4)	0.049 (3)	0.008 (3)	0.007 (3)	0.003 (3)
C18	0.063 (3)	0.053 (3)	0.040 (3)	0.001 (3)	0.000 (2)	0.004 (2)
C19	0.078 (4)	0.066 (4)	0.055 (3)	−0.012 (3)	−0.002 (3)	0.003 (3)
C20	0.076 (5)	0.089 (5)	0.075 (5)	−0.017 (4)	−0.004 (4)	0.012 (4)
C21	0.072 (5)	0.112 (7)	0.068 (4)	0.000 (5)	0.003 (4)	0.026 (5)
O21	0.042 (13)	0.14 (3)	0.12 (2)	−0.019 (14)	0.018 (13)	0.00 (2)
C22	0.079 (5)	0.095 (6)	0.070 (4)	0.019 (4)	0.013 (4)	0.013 (4)
C23	0.073 (4)	0.062 (4)	0.064 (4)	0.004 (3)	0.004 (3)	0.006 (3)

Geometric parameters (Å, º)

Fe1—C15	2.033 (8)	C7—C18	1.527 (8)
Fe1—C10	2.034 (7)	C7—C1	1.529 (8)
Fe1—C16	2.035 (7)	C7—H7	0.9800
Fe1—C11	2.037 (7)	C1—C2	1.375 (9)
Fe1—C9	2.040 (7)	C1—C6	1.387 (9)
Fe1—C13	2.042 (7)	C2—C3	1.378 (10)
Fe1—C12	2.044 (7)	C2—H2	0.9300
Fe1—C14	2.047 (8)	C3—C4	1.367 (13)
Fe1—C17	2.048 (7)	C3—H3	0.9300
Fe1—C8	2.058 (6)	C4—O4	1.366 (9)
C8—C9	1.423 (9)	C4—C5	1.373 (12)
C8—C12	1.435 (8)	C4—H4	0.9300
C8—C7	1.497 (8)	O4—H4O	0.8200
C9—C10	1.429 (10)	C5—C6	1.380 (9)

C9—H9	0.9300	C5—H5	0.9300
C10—C11	1.392 (12)	C6—H6	0.9300
C10—H10	0.9300	C18—C23	1.372 (9)
C11—C12	1.418 (10)	C18—C19	1.396 (9)
C11—H11	0.9300	C19—C20	1.374 (11)
C12—H12	0.9300	C19—H19	0.9300
C13—C14	1.396 (13)	C20—C21	1.365 (13)
C13—C17	1.400 (11)	C20—H20	0.9300
C13—H13	0.9300	C21—O21	1.28 (2)
C14—C15	1.435 (14)	C21—C22	1.376 (13)
C14—H14	0.9300	C21—H21	0.9300
C15—C16	1.411 (12)	O21—H21O	0.8200
C15—H15	0.9300	C22—C23	1.389 (11)
C16—C17	1.396 (10)	C22—H22	0.9300
C16—H16	0.9300	C23—H23	0.9300
C17—H17	0.9300		

C15—Fe1—C10	116.2 (3)	C17—C13—Fe1	70.2 (4)
C15—Fe1—C16	40.6 (4)	C14—C13—H13	125.8
C10—Fe1—C16	149.3 (3)	C17—C13—H13	125.8
C15—Fe1—C11	106.5 (3)	Fe1—C13—H13	125.4
C10—Fe1—C11	40.0 (3)	C13—C14—C15	107.9 (7)
C16—Fe1—C11	116.7 (3)	C13—C14—Fe1	69.9 (5)
C15—Fe1—C9	150.2 (3)	C15—C14—Fe1	68.9 (5)
C10—Fe1—C9	41.1 (3)	C13—C14—H14	126.0
C16—Fe1—C9	168.4 (3)	C15—C14—H14	126.0
C11—Fe1—C9	68.7 (3)	Fe1—C14—H14	126.8
C15—Fe1—C13	68.3 (4)	C16—C15—C14	106.6 (7)
C10—Fe1—C13	130.6 (4)	C16—C15—Fe1	69.8 (4)
C16—Fe1—C13	67.6 (3)	C14—C15—Fe1	69.9 (5)
C11—Fe1—C13	167.3 (4)	C16—C15—H15	126.7
C9—Fe1—C13	109.6 (3)	C14—C15—H15	126.7
C15—Fe1—C12	127.8 (3)	Fe1—C15—H15	125.2
C10—Fe1—C12	67.8 (3)	C17—C16—C15	108.7 (7)
C16—Fe1—C12	108.1 (3)	C17—C16—Fe1	70.5 (4)
C11—Fe1—C12	40.7 (3)	C15—C16—Fe1	69.6 (5)
C9—Fe1—C12	68.6 (3)	C17—C16—H16	125.7
C13—Fe1—C12	151.6 (3)	C15—C16—H16	125.7
C15—Fe1—C14	41.2 (4)	Fe1—C16—H16	125.8
C10—Fe1—C14	108.8 (3)	C16—C17—C13	108.4 (7)
C16—Fe1—C14	68.0 (3)	C16—C17—Fe1	69.5 (4)
C11—Fe1—C14	128.7 (4)	C13—C17—Fe1	69.8 (4)
C9—Fe1—C14	117.7 (4)	C16—C17—H17	125.8
C13—Fe1—C14	39.9 (4)	C13—C17—H17	125.8
C12—Fe1—C14	167.0 (4)	Fe1—C17—H17	126.5
C15—Fe1—C17	67.9 (3)	C8—C7—C18	111.0 (5)
C10—Fe1—C17	169.2 (3)	C8—C7—C1	114.1 (5)
C16—Fe1—C17	40.0 (3)	C18—C7—C1	111.6 (5)
C11—Fe1—C17	150.3 (3)	C8—C7—H7	106.6
C9—Fe1—C17	130.8 (3)	C18—C7—H7	106.6
C13—Fe1—C17	40.0 (3)	C1—C7—H7	106.6
C12—Fe1—C17	118.4 (3)	C2—C1—C6	117.3 (6)

C14—Fe1—C17	67.2 (3)	C2—C1—C7	120.9 (6)
C15—Fe1—C8	167.1 (4)	C6—C1—C7	121.9 (6)
C10—Fe1—C8	68.3 (3)	C1—C2—C3	122.1 (7)
C16—Fe1—C8	129.8 (3)	C1—C2—H2	118.9
C11—Fe1—C8	68.7 (3)	C3—C2—H2	118.9
C9—Fe1—C8	40.6 (2)	C4—C3—C2	119.2 (7)
C13—Fe1—C8	118.8 (3)	C4—C3—H3	120.4
C12—Fe1—C8	41.0 (2)	C2—C3—H3	120.4
C14—Fe1—C8	150.9 (4)	O4—C4—C3	116.0 (9)
C17—Fe1—C8	109.9 (3)	O4—C4—C5	123.3 (9)
C9—C8—C12	107.3 (6)	C3—C4—C5	120.7 (6)
C9—C8—C7	128.5 (6)	C3—C4—H4	119.6
C12—C8—C7	124.0 (6)	C5—C4—H4	119.6
C9—C8—Fe1	69.0 (4)	C4—O4—H4O	109.5
C12—C8—Fe1	69.0 (4)	C4—C5—C6	119.2 (7)
C7—C8—Fe1	131.5 (4)	C4—C5—H5	120.4
C8—C9—C10	107.3 (7)	C6—C5—H5	120.4
C8—C9—Fe1	70.3 (4)	C5—C6—C1	121.6 (7)
C10—C9—Fe1	69.2 (4)	C5—C6—H6	119.2
C8—C9—H9	126.3	C1—C6—H6	119.2
C10—C9—H9	126.3	C23—C18—C19	118.8 (6)
Fe1—C9—H9	125.7	C23—C18—C7	122.6 (6)
C11—C10—C9	109.2 (7)	C19—C18—C7	118.6 (6)
C11—C10—Fe1	70.1 (4)	C20—C19—C18	120.8 (8)
C9—C10—Fe1	69.7 (4)	C20—C19—H19	119.6
C11—C10—H10	125.4	C18—C19—H19	119.6
C9—C10—H10	125.4	C21—C20—C19	120.0 (8)
Fe1—C10—H10	126.4	C21—C20—H20	120.0
C10—C11—C12	108.1 (6)	C19—C20—H20	120.0
C10—C11—Fe1	69.9 (4)	O21—C21—C20	129.1 (19)
C12—C11—Fe1	69.9 (4)	O21—C21—C22	110.8 (18)
C10—C11—H11	126.0	C20—C21—C22	120.1 (8)
C12—C11—H11	126.0	C20—C21—H21	120.0
Fe1—C11—H11	125.8	C22—C21—H21	120.0
C11—C12—C8	108.2 (7)	C21—O21—H21O	109.5
C11—C12—Fe1	69.4 (4)	C21—C22—C23	120.3 (8)
C8—C12—Fe1	70.0 (4)	C21—C22—H22	119.9
C11—C12—H12	125.9	C23—C22—H22	119.9
C8—C12—H12	125.9	C18—C23—C22	120.1 (7)
Fe1—C12—H12	126.2	C18—C23—H23	120.0
C14—C13—C17	108.4 (7)	C22—C23—H23	120.0
C14—C13—Fe1	70.2 (5)		

C8—C7—C18—C23	−28.7 (7)	C8—C7—C1—C2	−108.2 (7)
C8—C7—C18—C19	150.4 (5)	C8—C7—C1—C6	71.9 (8)
