# Supplementary material for: Trapping para-Quinone Methide Intermediates with Ferrocene: Synthesis and Preliminary Biological Evaluation of New Phenol-Ferrocene Conjugates
Source: Molecules. 2018 Jun 1;23(6):1335. doi: 10.3390/molecules23061335 (PMC6099632; doi:10.3390/molecules23061335)
Supplement: Supplementary file 1 [file molecules-23-01335-s001.zip › Supplementary Files/Supporting Material.pdf]

# Supplementary Materials

# Trapping *para*-Quinone Methide Intermediates with Ferrocene: Synthesis and Preliminary Biological Evaluation of New Phenol-Ferrocene Conjugates

Silvia González-Pelayo, Enol López, Javier Borge, Noemí de-los-Santos-Álvarez and Luis A. López*

**TABLE OF CONTENTS:**


| | |
|---|---|
| **Figure S1** | $^{1}$H-NMR (300 MHz, $CDCl_3$) and $^{13}$C-NMR (75 MHz, $CDCl_3$) spectra of ferrocene derivative **3a**. |
| **Figure S2** | $^{1}$H-NMR (300 MHz, $CDCl_3$) and $^{13}$C-NMR (75 MHz, $CDCl_3$) spectra of ferrocene derivative **3b**. |
| **Figure S3** | $^{1}$H-NMR (300 MHz, $CDCl_3$) and $^{13}$C-NMR (75 MHz, $CDCl_3$) spectra of ferrocene derivative **3c**. |
| **Figure S4** | $^{1}$H-NMR (300 MHz, $CDCl_3$) and $^{13}$C-NMR (75 MHz, $CDCl_3$) spectra of ferrocene derivative **3d**. |
| **Figure S5** | $^{1}$H-NMR (300 MHz, $CDCl_3$) and $^{13}$C-NMR (75 MHz, $CDCl_3$) spectra of ferrocene derivative **3e**. |
| **Figure S6** | $^{1}$H-NMR (300 MHz, $CDCl_3$) and $^{13}$C-NMR (75 MHz, $CDCl_3$) spectra of ferrocene derivative **3f**. |
| **Figure S7** | $^{1}$H-NMR (300 MHz, $CDCl_3$) and $^{13}$C-NMR (75 MHz, $CDCl_3$) spectra of ferrocene derivative **3g**. |
| **Figure S8** | $^{1}$H-NMR (300 MHz, $CDCl_3$) and $^{13}$C-NMR (75 MHz, $CDCl_3$) spectra of ferrocene derivative **3h**. |
| **Figure S9** | $^{1}$H-NMR (300 MHz, $CDCl_3$) and $^{13}$C-NMR (75 MHz, $CDCl_3$) spectra of ferrocene derivative **3i**. |
| **Figure S10** | $^{1}$H-NMR (300 MHz, $CDCl_3$) and $^{13}$C-NMR (75 MHz, $CDCl_3$) spectra of ferrocene derivative **3j**. |
| **Figure S11** | $^{1}$H-NMR (300 MHz, $CDCl_3$) and $^{13}$C-NMR (75 MHz, $CDCl_3$) spectra of ferrocene derivative **3k**. |
| **Figure S12** | $^{1}$H-NMR (300 MHz, $CDCl_3$) and $^{13}$C-NMR (75 MHz, $CDCl_3$) spectra of ferrocene derivative **3l**. |
| **Figure S13** | $^{1}$H-NMR (300 MHz, $CDCl_3$) and $^{13}$C-NMR (75 MHz, $CDCl_3$) spectra of ferrocene derivative **5**. |

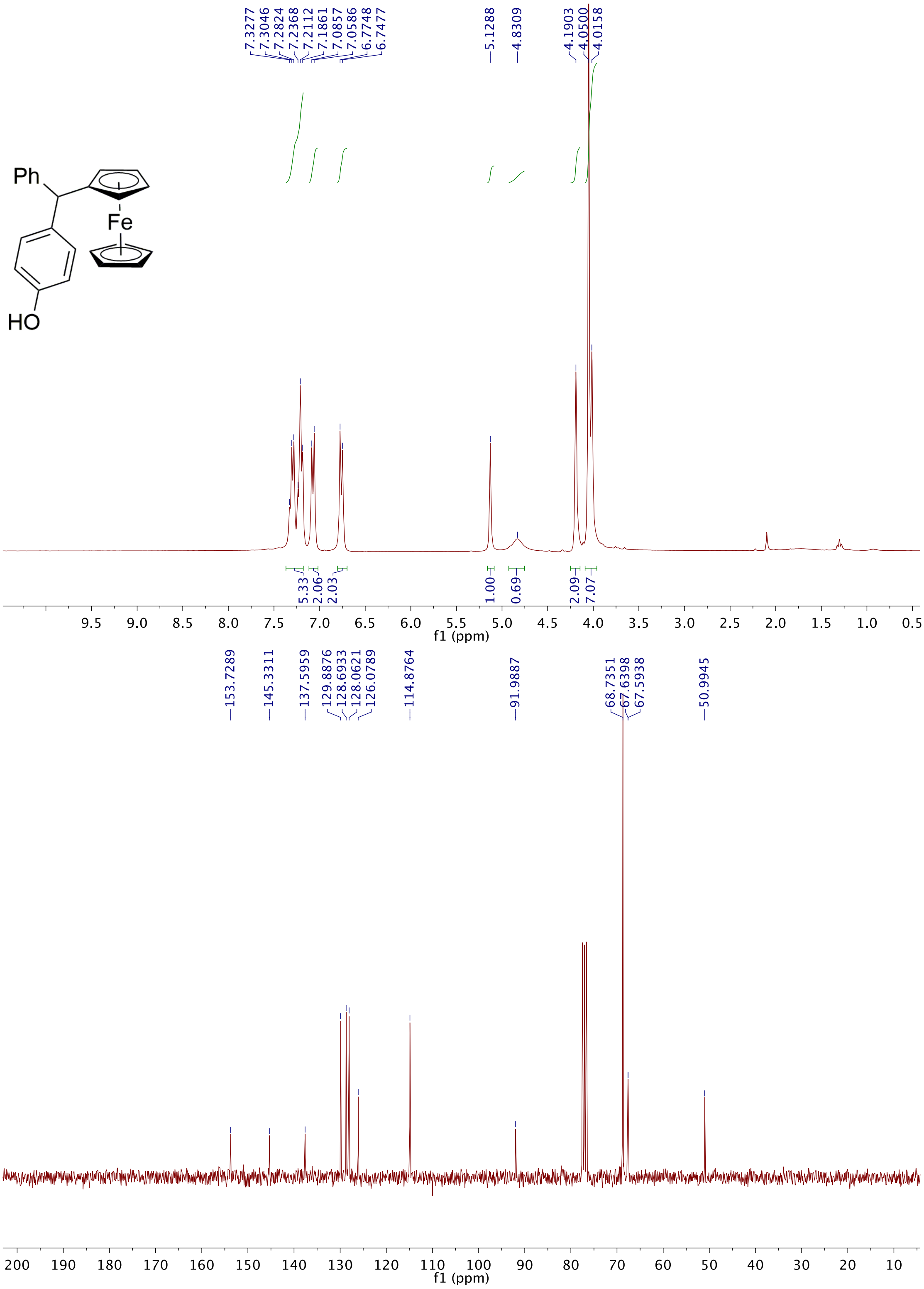


**Figure S1**. $^{1}$H-NMR (300 MHz, $CDCl_3$) and $^{13}$C-NMR (75 MHz, $CDCl_3$) spectra of ferrocene derivative **3a**.

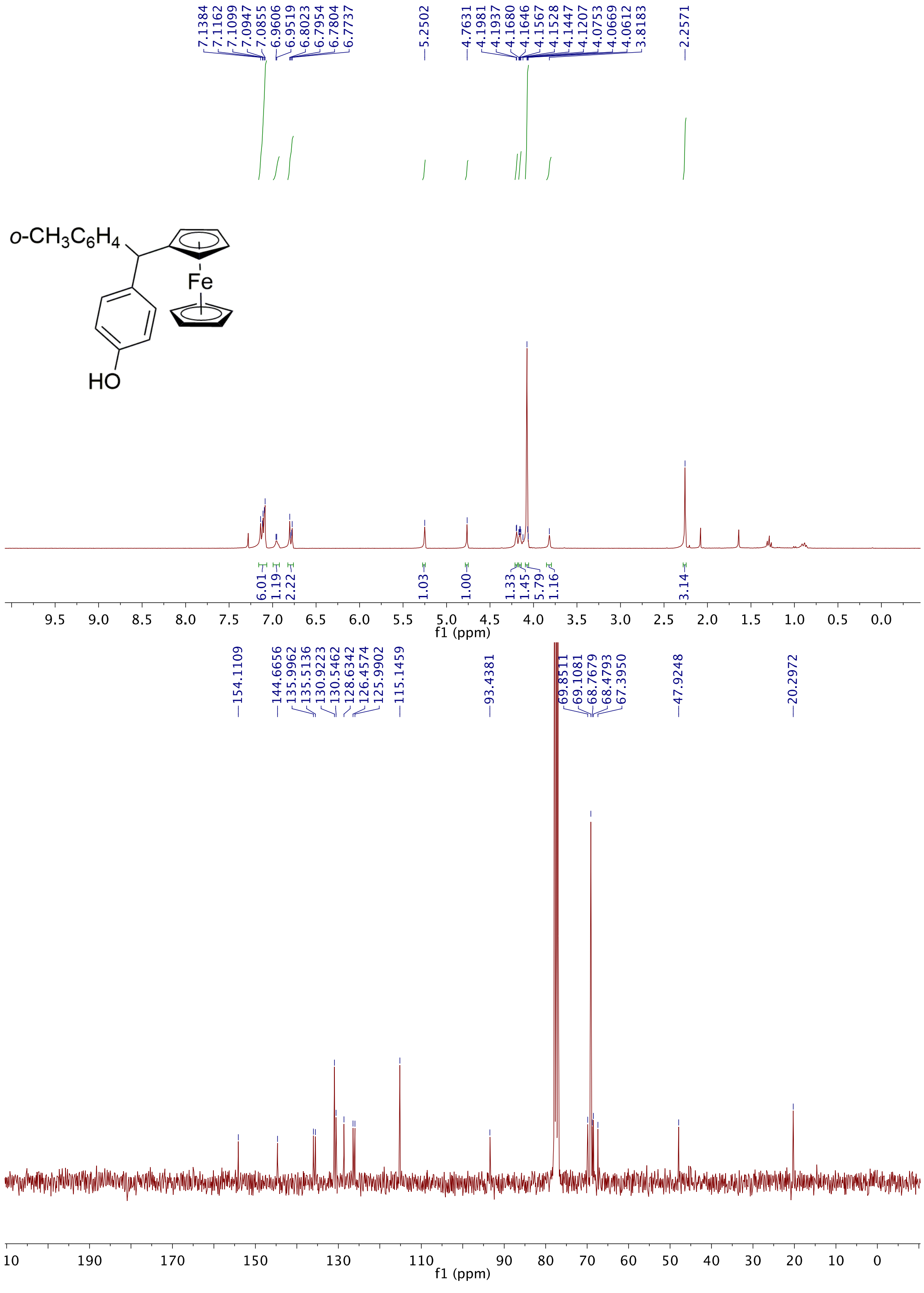


**Figure S2**. $^{1}H$-NMR (300 MHz, $CDCl_3$) and $^{13}C$-NMR (75 MHz, $CDCl_3$) spectra of ferrocene derivative **3b**.

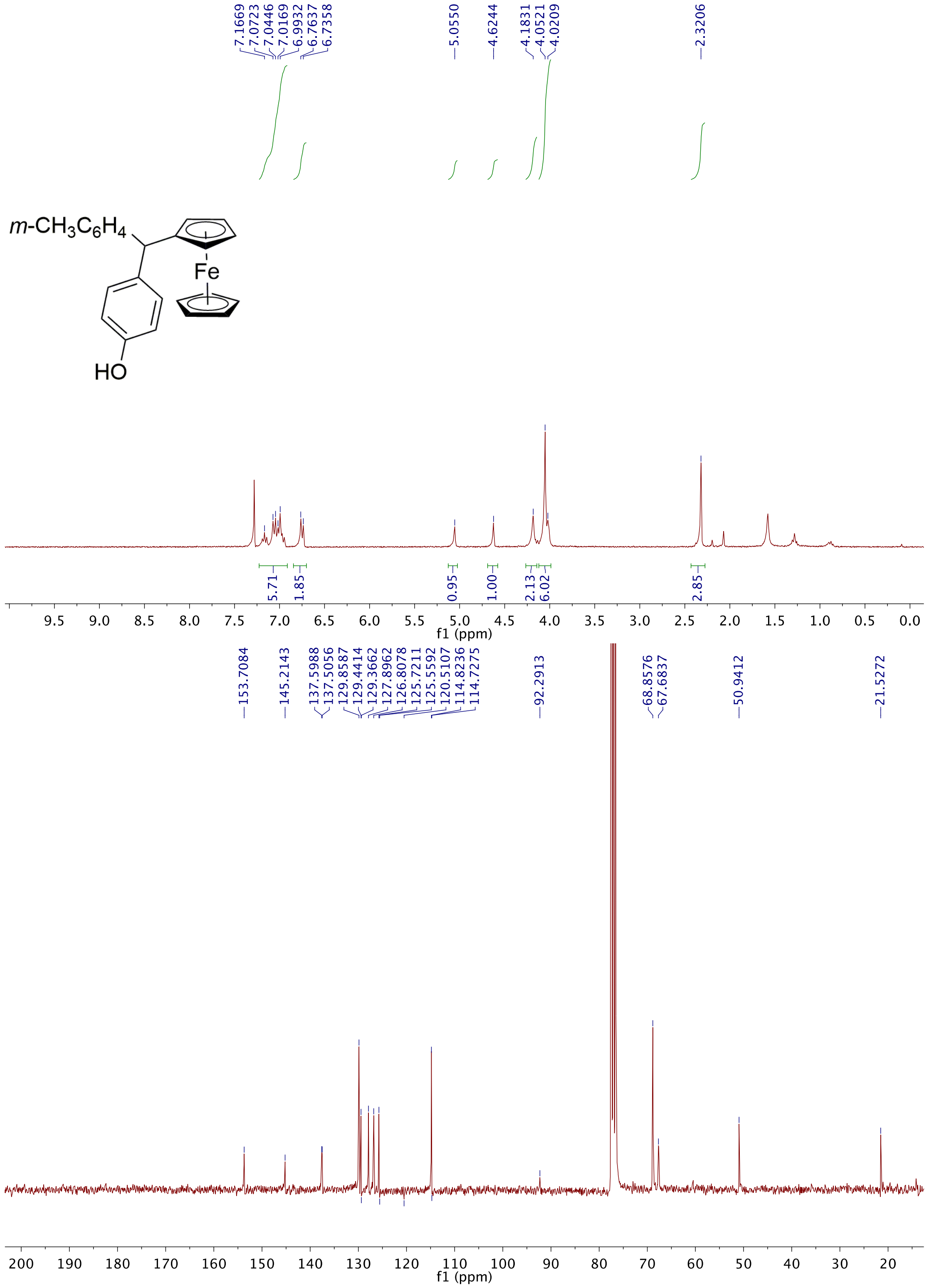


**Figure S3**. $^{1}H$-NMR (300 MHz, $CDCl_3$) and $^{13}C$-NMR (75 MHz, $CDCl_3$) spectra of ferrocene derivative **3c**.

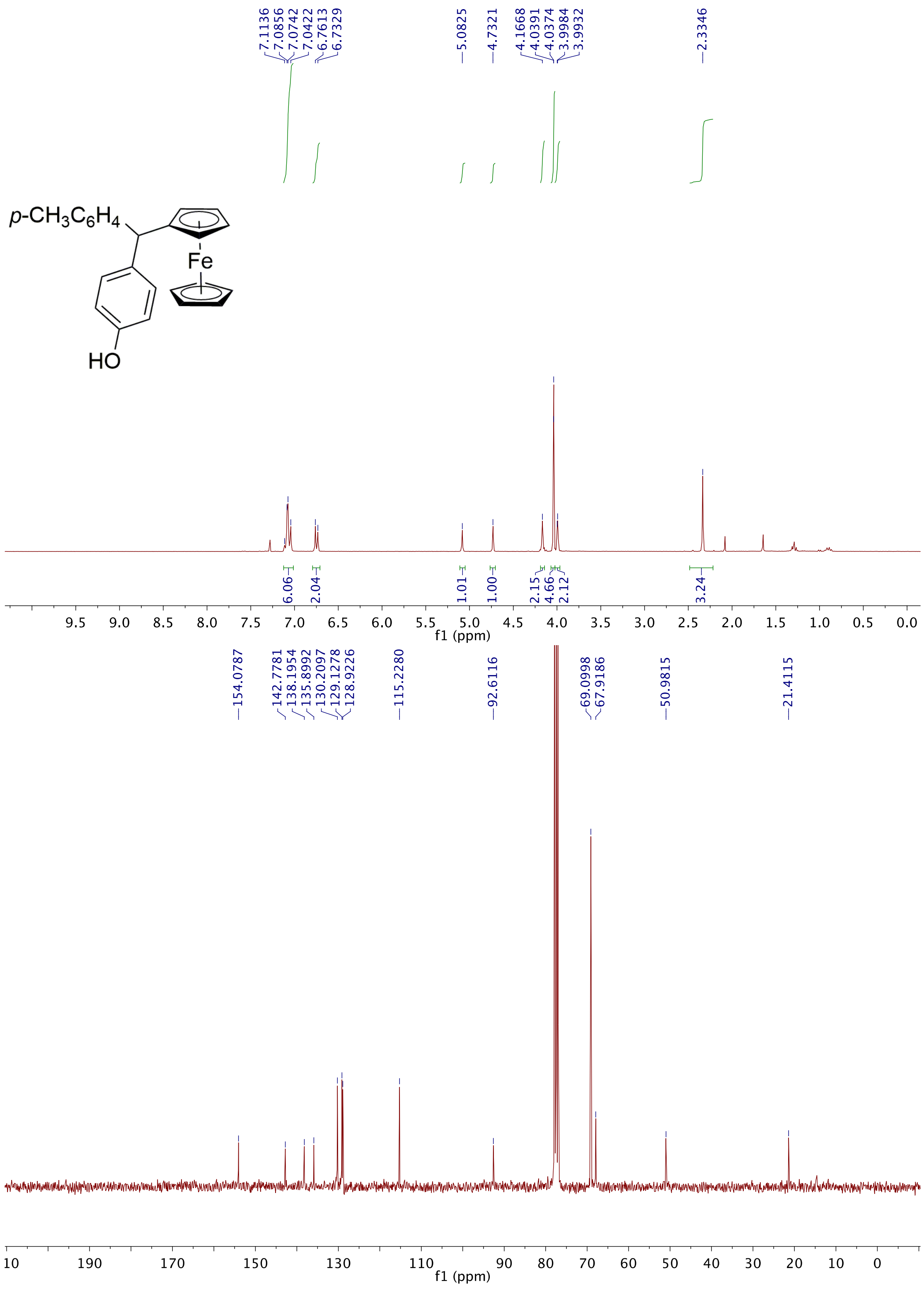


**Figure S4**. $^{1}H$-NMR (300 MHz, $CDCl_3$) and $^{13}C$-NMR (75 MHz, $CDCl_3$) spectra of ferrocene derivative **3d**.

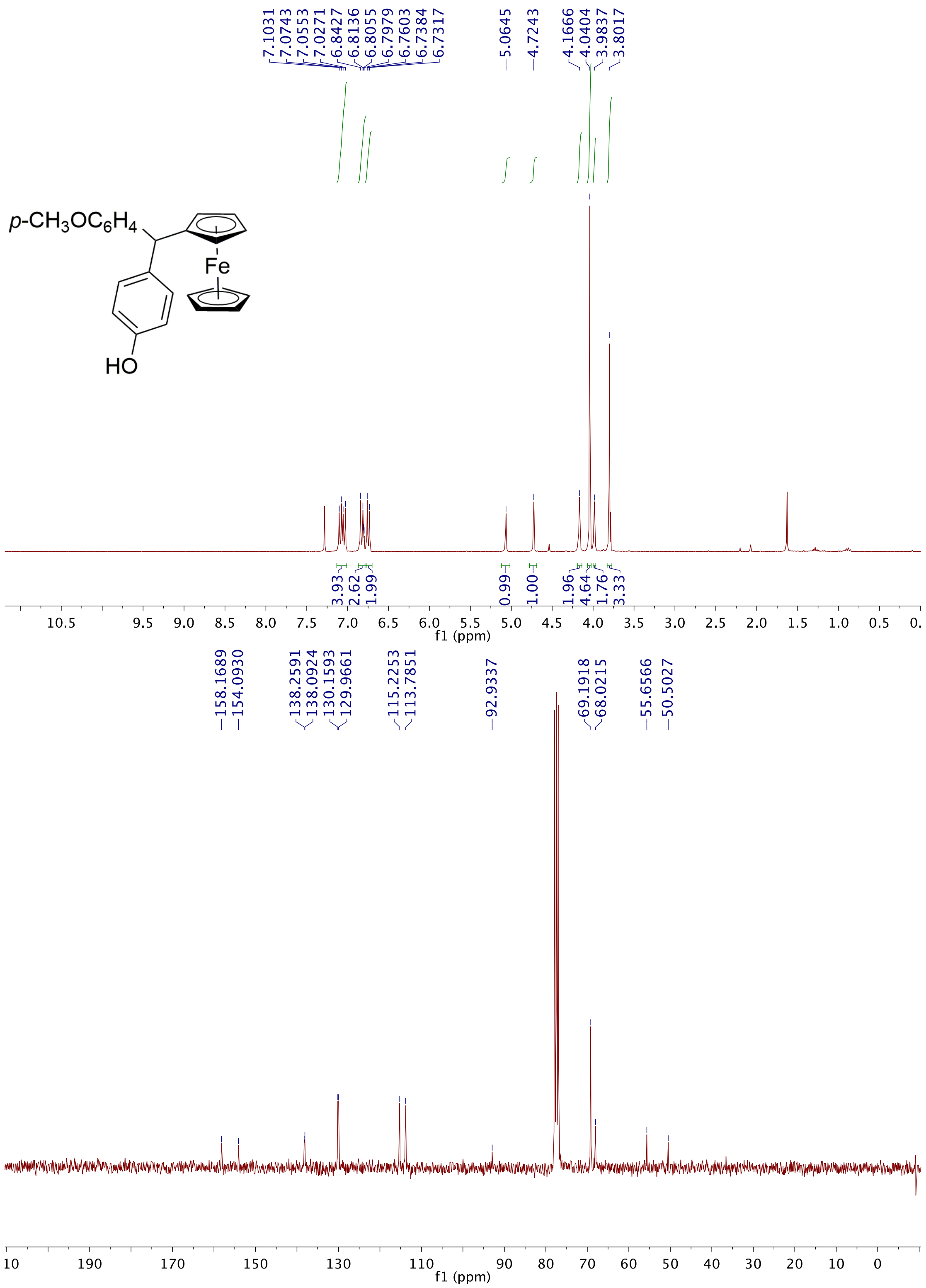


**Figure S5**. $^{1}H$-NMR (300 MHz, $CDCl_3$) and $^{13}C$-NMR (75 MHz, $CDCl_3$) spectra of ferrocene derivative **3e**.

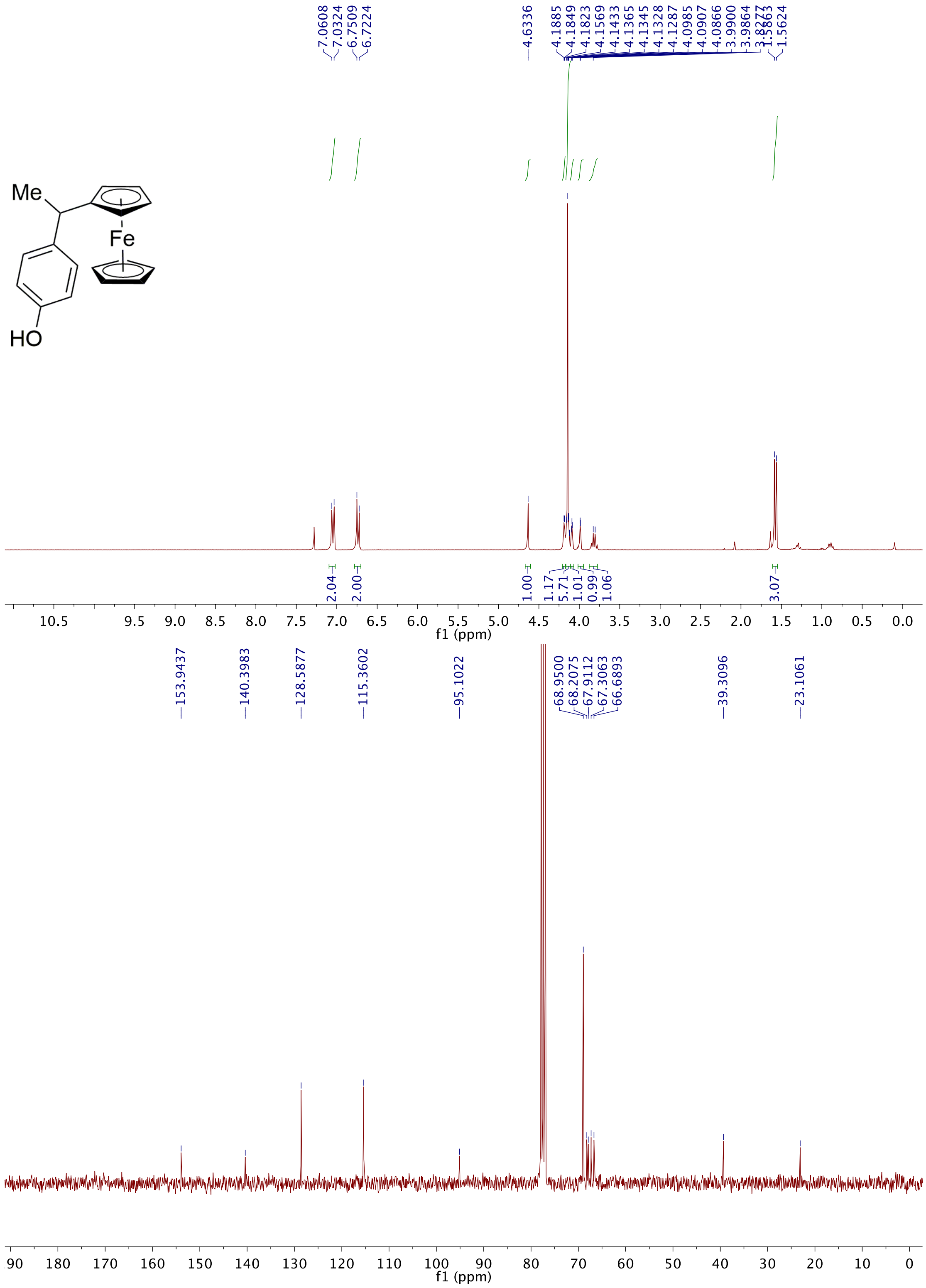


**Figure S6**. $^1$H-NMR (300 MHz, $CDCl_3$) and $^{13}$C-NMR (75 MHz, $CDCl_3$) spectra of ferrocene derivative **3f**.

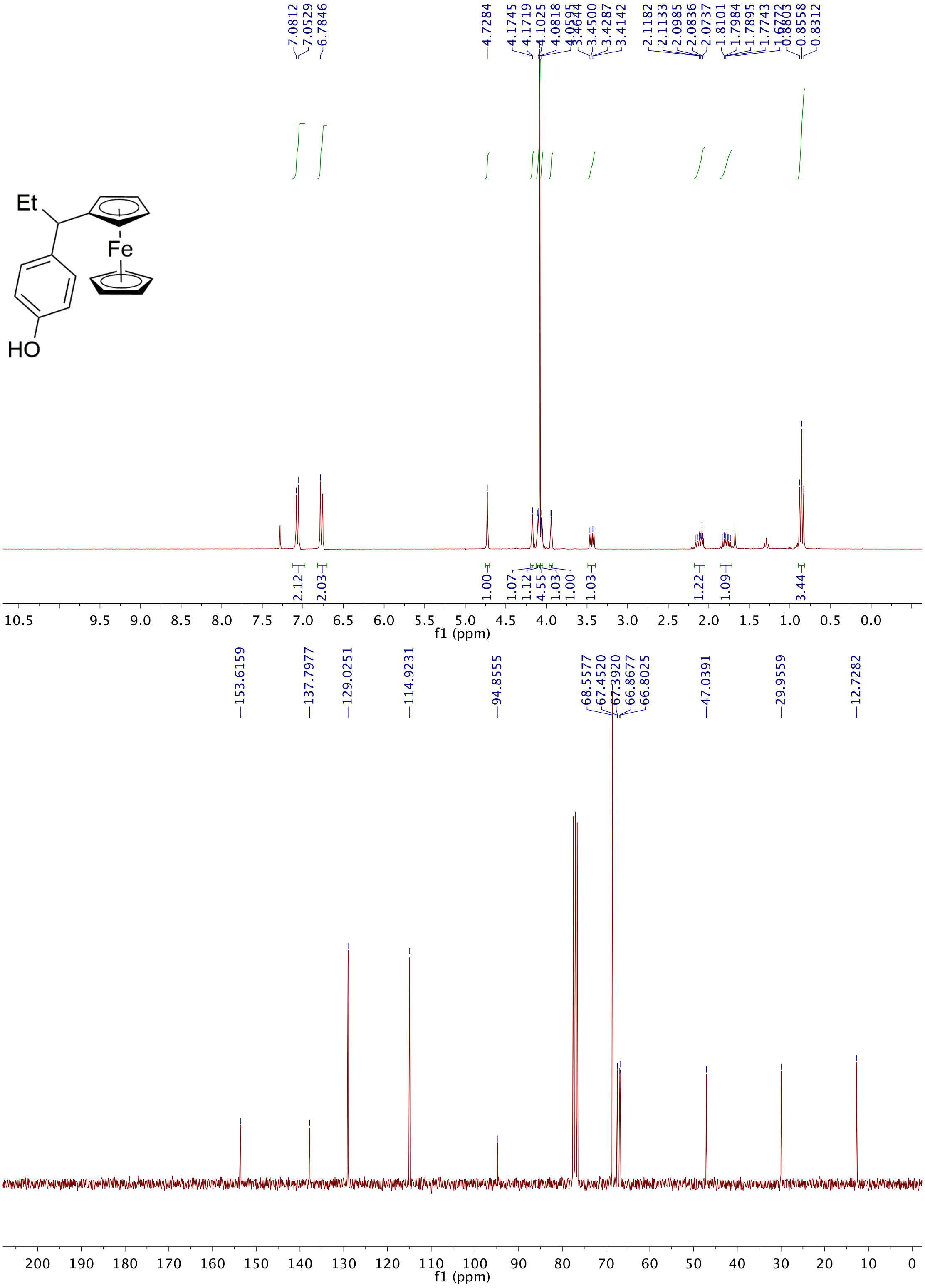


**Figure S7**. $^{1}$H-NMR (300 MHz, $CDCl_3$) and $^{13}$C-NMR (75 MHz, $CDCl_3$) spectra of ferrocene derivative **3g**.

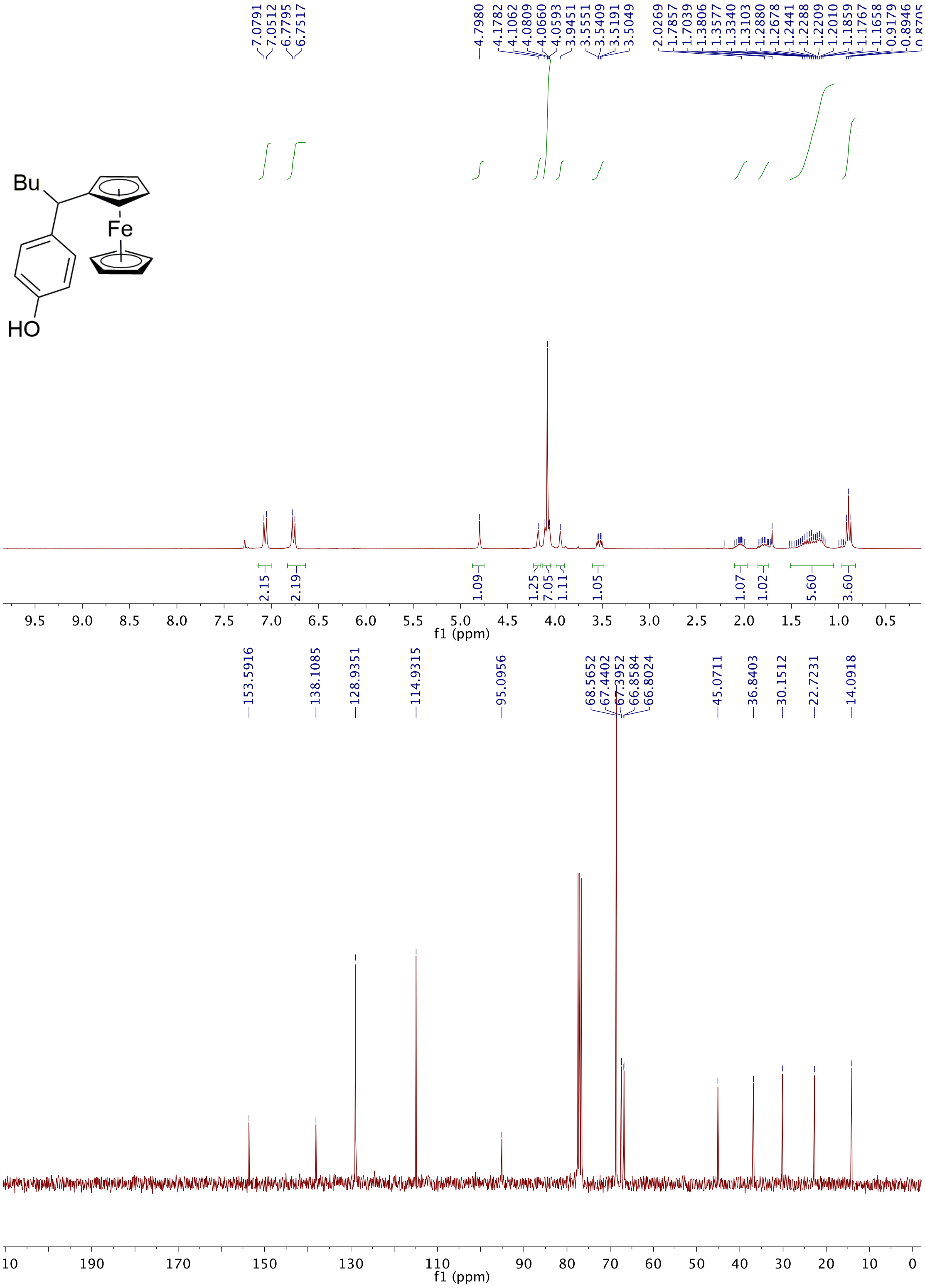


**Figure S8**. $^{1}H$-NMR (300 MHz, $CDCl_3$) and $^{13}C$-NMR (75 MHz, $CDCl_3$) spectra of ferrocene derivative **3h**.

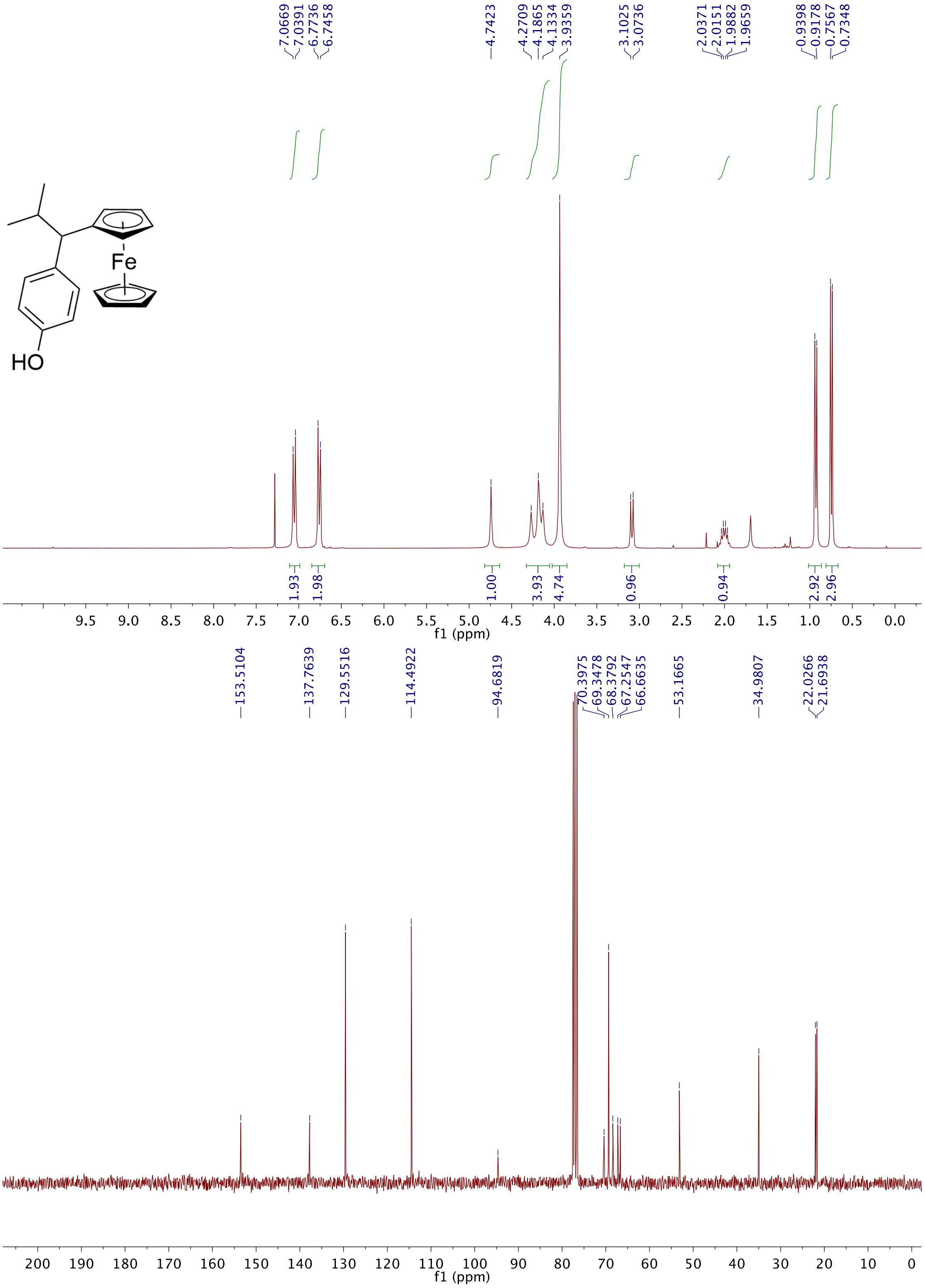


**Figure S9**. $^{1}H$-NMR (300 MHz, $CDCl_3$) and $^{13}C$-NMR (75 MHz, $CDCl_3$) spectra of ferrocene derivative **3i**.

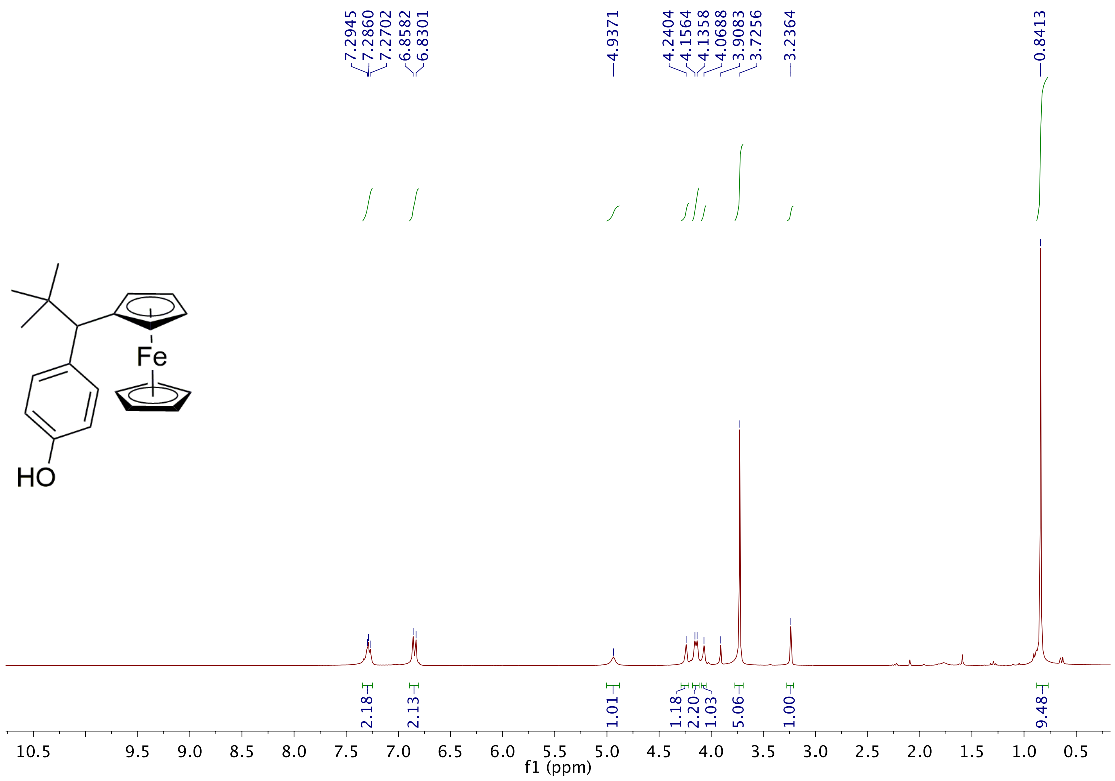

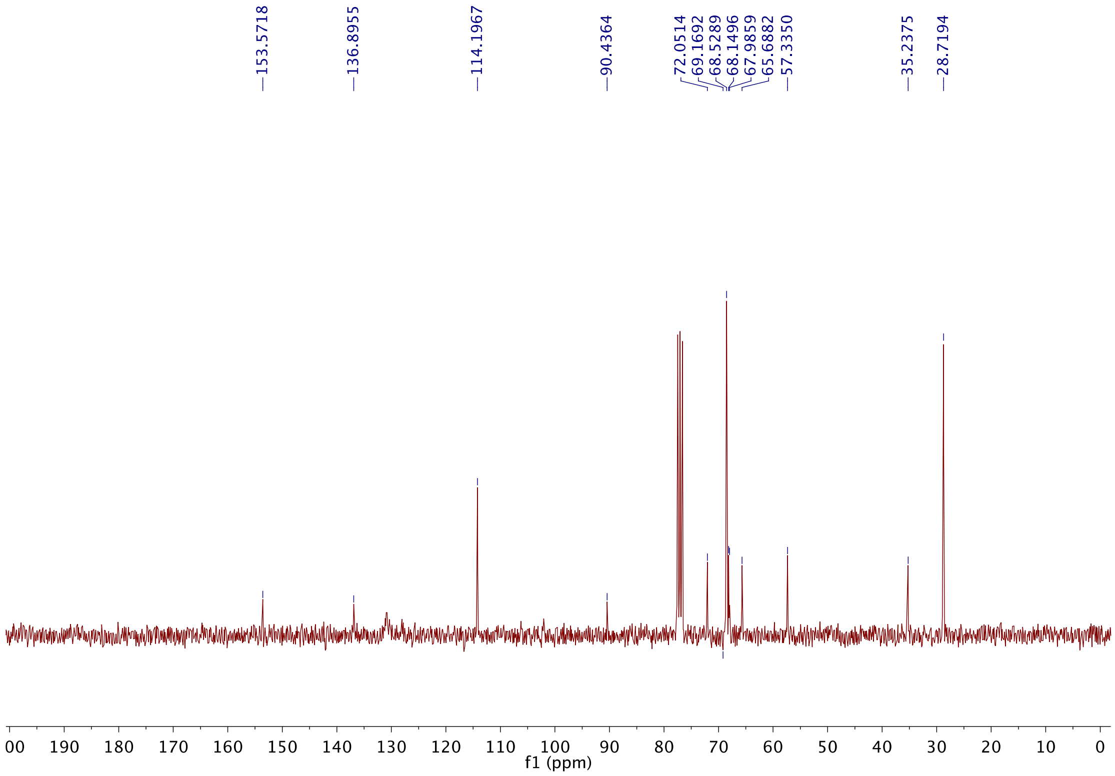

**Figure S10**. $^{1}$H-NMR (300 MHz, $CDCl_3$) and $^{13}$C-NMR (75 MHz, $CDCl_3$) spectra of ferrocene derivative **3j**.

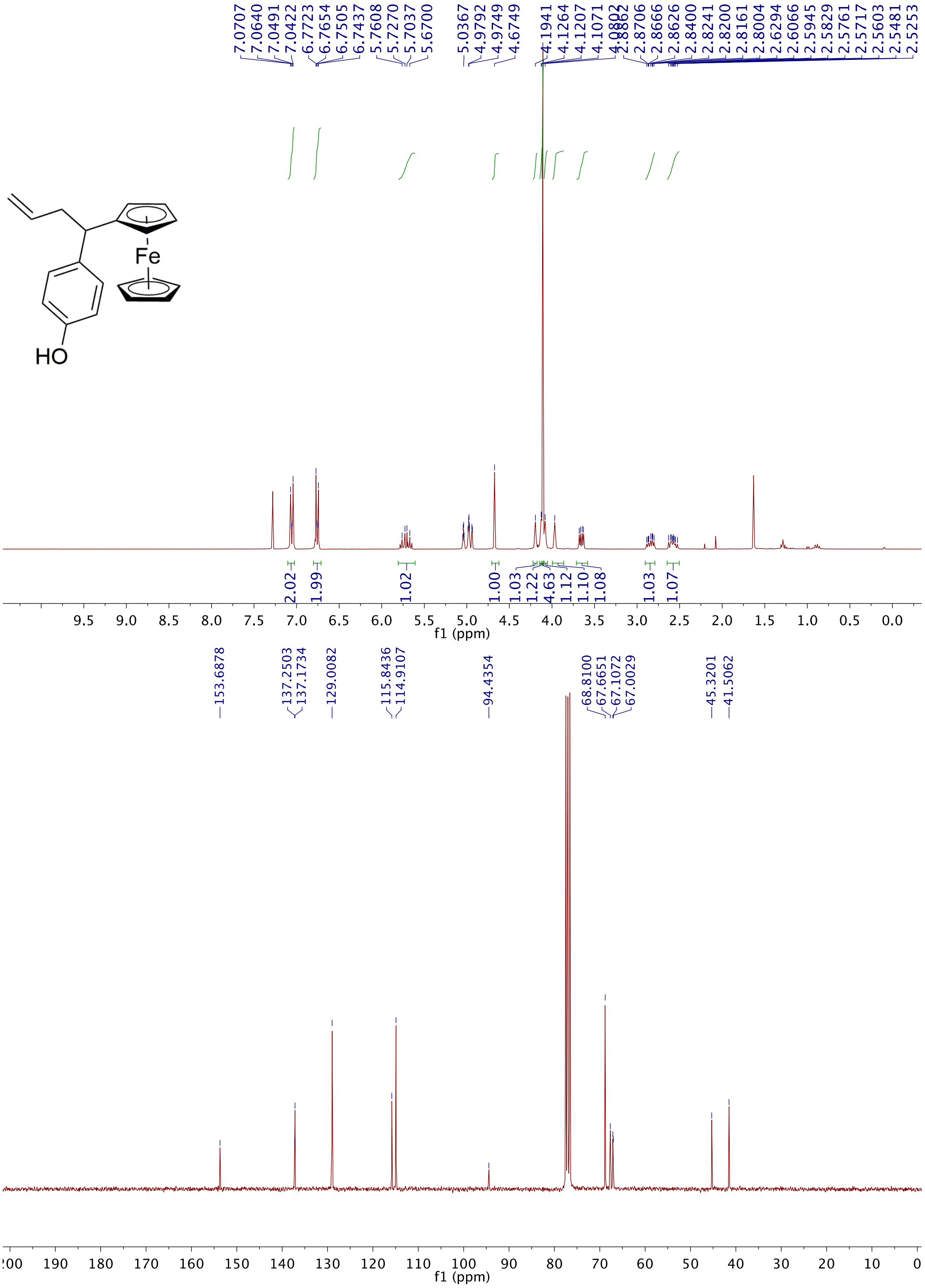


**Figure S11**. $^1H$-NMR (300 MHz, $CDCl_3$) and $^{13}C$-NMR (75 MHz, $CDCl_3$) spectra of ferrocene derivative **3k**.

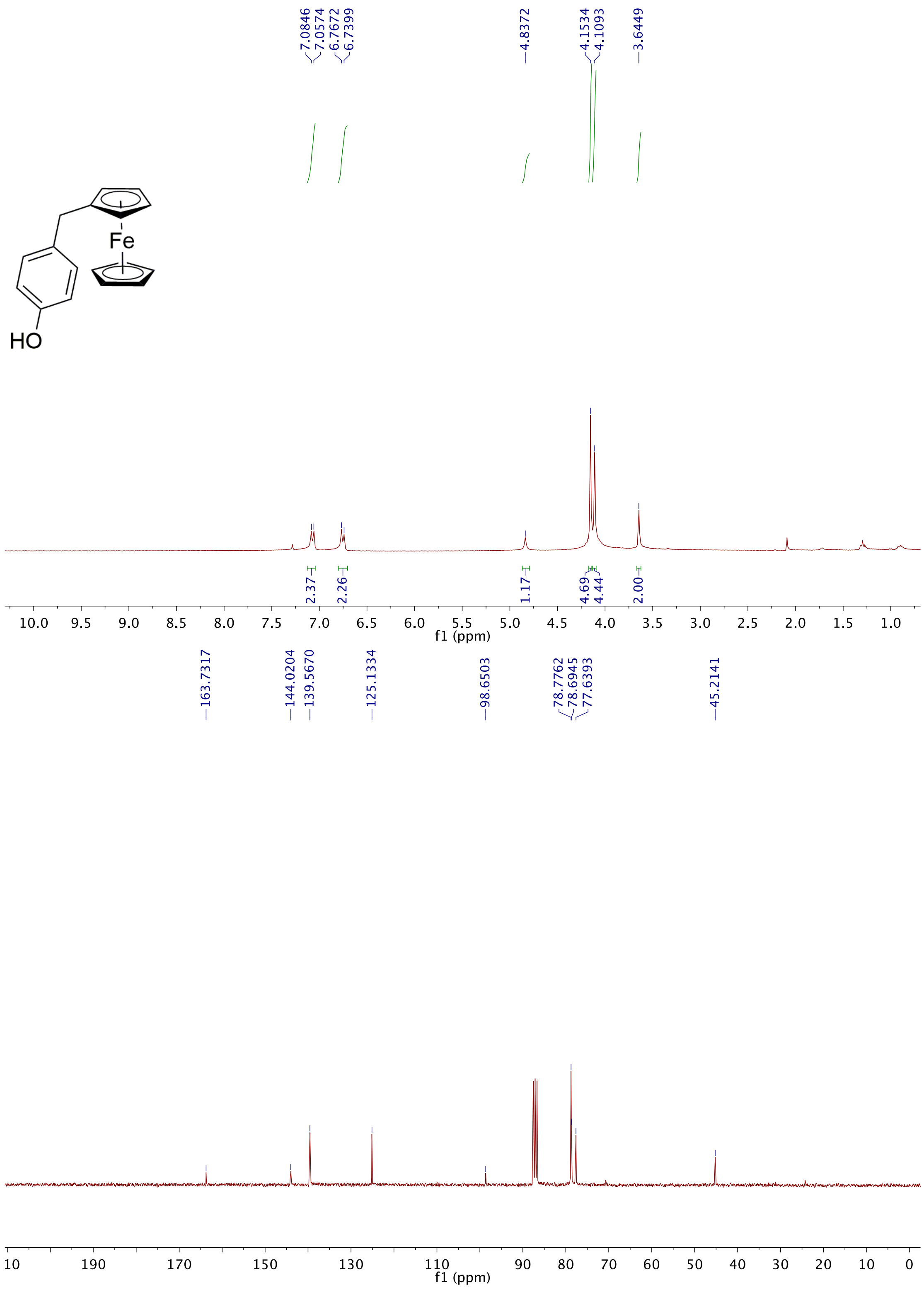


**Figure S12**. $^{1}$H-NMR (300 MHz, $CDCl_3$) and $^{13}$C-NMR (75 MHz, $CDCl_3$) spectra of ferrocene derivative **3l**.

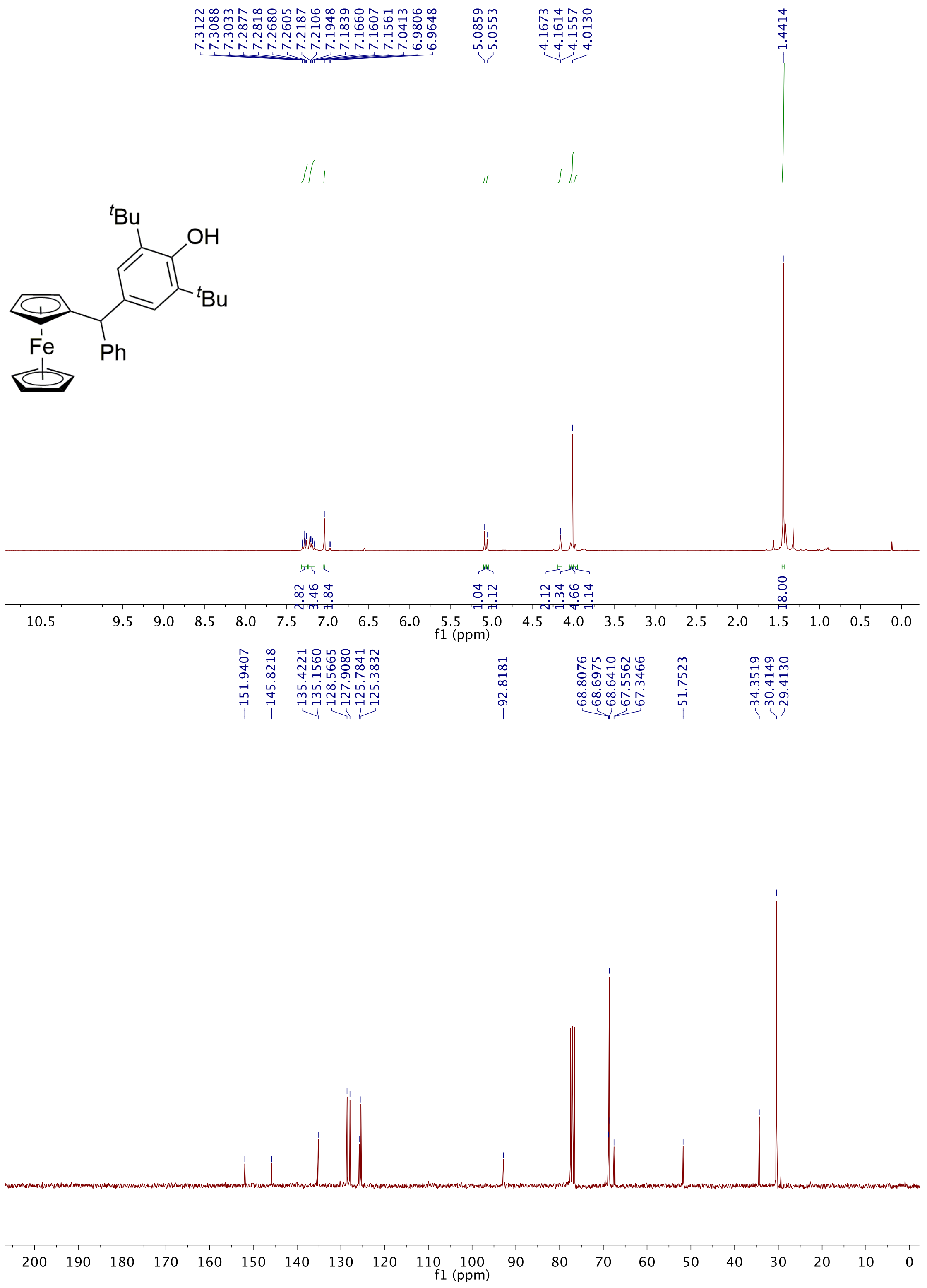


**Figure S13**. $^{1}$H-NMR (300 MHz, $CDCl_3$) and $^{13}$C-NMR (75 MHz, $CDCl_3$) spectra of ferrocene derivative **5**.
